# Genome-wide assessment of the carriers involved in the cellular uptake of drugs: a model system in yeast

**DOI:** 10.1186/1741-7007-9-70

**Published:** 2011-10-24

**Authors:** Karin Lanthaler, Elizabeth Bilsland, Paul D Dobson, Harry J Moss, Pınar Pir, Douglas B Kell, Stephen G Oliver

**Affiliations:** 1 School of Chemistry, University of Manchester, Oxford Road, Manchester, M13 9PL, UK; 2 Manchester Interdisciplinary Biocentre, 131 Princess Street, Manchester, M1 7DN, UK; 3 Faculty of Life Sciences, University of Manchester, Michael Smith Building, Oxford Road, Manchester, M13 9PT, UK; 4 Cambridge Systems Biology Centre and Department of Biochemistry, University of Cambridge, Sanger Building, 80 Tennis Court Road, Cambridge, CB2 1GA, UK

## Abstract

**Background:**

The uptake of drugs into cells has traditionally been considered to be predominantly via passive diffusion through the bilayer portion of the cell membrane. The recent recognition that drug uptake is mostly carrier-mediated raises the question of which drugs use which carriers.

**Results:**

To answer this, we have constructed a chemical genomics platform built upon the yeast gene deletion collection, using competition experiments in batch fermenters and robotic automation of cytotoxicity screens, including protection by 'natural' substrates. Using these, we tested 26 different drugs and identified the carriers required for 18 of the drugs to gain entry into yeast cells.

**Conclusions:**

As well as providing a useful platform technology, these results further substantiate the notion that the cellular uptake of pharmaceutical drugs normally occurs via carrier-mediated transport and indicates that establishing the identity and tissue distribution of such carriers should be a major consideration in the design of safe and effective drugs.

## Background

Of the many reasons for the attrition of candidate drugs during the development process, toxicity or lack of efficacy *in vivo *are among the most frequent [1,2]. Excessive concentration in particular tissues can be the cause of the former, while failure to reach targets can contribute to the latter. The steady-state tissue distributions of drugs are determined by the rates of their uptake and efflux. While the role of carriers as mediators of drug efflux is well appreciated, uptake was, until recently, considered to be almost entirely a process of passive diffusion through the lipid part of the membrane and therefore largely determined by drug lipophilicity, with carrier uptake considered exceptional [3]. It is now increasingly recognized that drug uptake is predominantly carrier-mediated [4-7]. The missing information required to understand the tissue distributions of drugs is thus represented by the specificities and location of uptake carriers. Although there are any number of specific examples [6], the first task is to establish general methods for determining which of the known carriers are most responsible for the cellular uptake of particular drugs, as a prelude to establishing the tissue distributions of the relevant carriers.

*Saccharomyces cerevisiae *is a well-understood and widely used model organism for chemical genomics studies [8-12]. Existing data regarding the interaction of yeast cells with drugs have brought up a number of cases in which changes in the activity of specific carriers increase or decrease the sensitivity of cells to xenobiotics, with the clear implication that such carriers effect the entry of these drugs into cells or their exit from them [9,13-15]. A particular benefit of *S. cerevisiae *is the availability of a barcoded series of deletion mutants [16], whose relative rates of growth/survival can be tested in competition experiments (for examples, see [9,17-20]). We therefore recognized that if a drug is toxic when present at a high concentration inside the cell, but requires the activity of a carrier to be taken up by the cell, a strain with no or reduced carrier activity should be relatively resistant to the drug and survive better in competition experiments when compared to strains with normal uptake activity. This analysis also predicts that if another non-toxic (and possibly 'natural' [21]) substrate for the carrier is known, then this will compete with the toxic drug for uptake into the wild-type (WT) strain (assuming equivalent binding sites), thereby conferring phenotypic protection against toxicity.

In the present work, we have employed two high-throughput platforms that exploit resistance associated with gene deletion to identify drug transporters. We have used these approaches to study the uptake of 26 pharmaceutically active (but - in yeast - cytotoxic) compounds. The first platform consists of parallelized screens where we grow the total pool of homozygous diploid yeast gene deletants in batch fermenters, with and without the drug. The proportions of the different strains in the population are assayed by amplifying their molecular barcodes and hybridizing them to a TAG4 oligonucleotide microarray. Resistant strains will account for an increasing proportion of the total pool in drug-treated compared to untreated conditions, because they are able to outcompete susceptible strains due to the resistance conferred by the gene deletion. The second platform screens strains individually and relies upon robotics to increase throughput by spotting strains deleted for genes encoding transporters onto 768-spot plates, allowing many strains to be screened in parallel.

These high-throughput experiments suggested uptake transporters for 18 of 26 compounds screened. For half of the compounds with suggested transporters, validation low-throughput experiments were performed confirming most of the suggested transporters. Furthermore, protection experiments using known ('native') substrates were performed for three of the drugs, confirming the role of the suggested transporter in drug uptake.

## Results

### Canavanine transport: a proof-of-principle experiment

**Figure 1:**
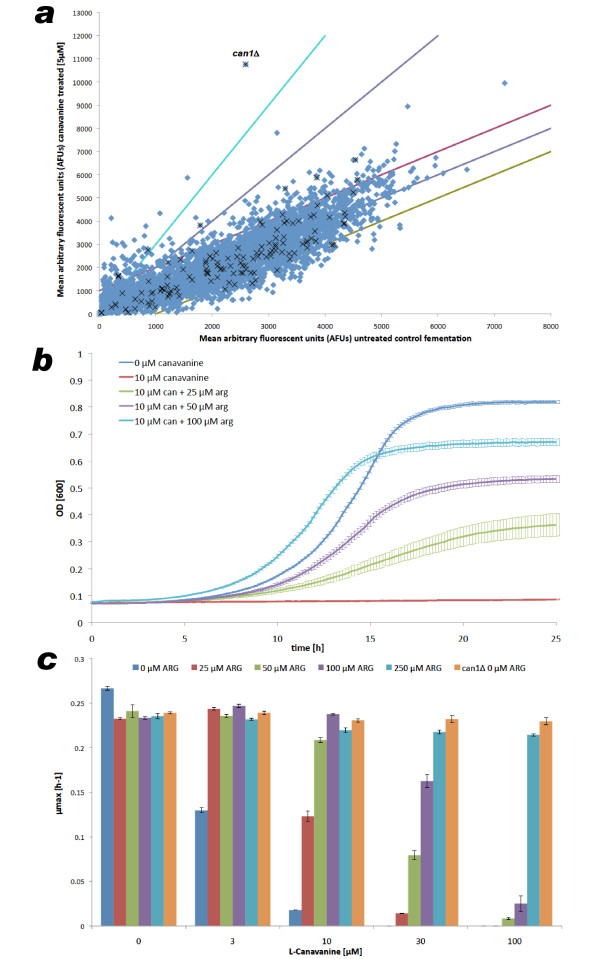
**Calibration and illustrative results from the pool competition approach**. **(a) **Pool competition results for selection for canavanine resistance. The abscissa indicates the proportion of each strain in the untreated pool. When treated (y-axis) the ability of the *can1*Δ/*can1*Δ diploid to resist canavanine confers a major growth advantage to the strain such that it outcompetes all others to become the most abundant strain. Pink and green lines (± 1000 y-translation of parity line) detail the boundary within which 98% of deletants are found when comparing untreated controls, so providing a noise estimate. Indigo and cyan lines indicate treatment/control ratios of 2 and 3. Blue diamonds denote all deletants and black stars identify strains deleted for transporter genes. AFU = mean arbitrary fluorescent units measured for the TAG4 arrays of treated (canavanine) and untreated (control) competitions between the pools of homozygous deletants. **(b) **Growth curves of wild type (ydl227cΔ/ydl227cΔ) yeast strains in the presence of 10 μM of canavanine and increasing concentration of the competitor arginine. **(c) **Comparison of the maximum specific growth rate achieved by the WT strain in the presence and absence of canavanine illustrates the cytotoxic effect of the drug. The protective effects of various concentrations of arginine, one of the native substrates of Can1p, are shown over the drug concentration range (0 mM to 100 mM). A similar growth rate advantage to deleting *CAN1 *is obtained by adding 250 μM of arginine. Error bars = standard error of the mean; n = 3.

To calibrate and validate our experimental methods, canavanine, a known antimetabolite substrate [22] of the uptake transporter arginine permease (Can1p) was used. Canavanine is an arginine analogue that is readily incorporated into proteins, producing a toxic effect. A concentration of the drug was used that was sufficient to reduce the growth rate of the WT strain by 90% (that is, the 90% inhibitory concentration; IC_90_). Figure 1a shows results of the pool experiment using canavanine, with resistance associated with the *can1*Δ/*can1*Δ deletant demonstrated by that strain's top-ranked position on the drug-treated axis. By plotting the mean arbitrary fluorescent units from untreated and canavanine-treated pools, we could clearly identify the *can1*Δ/*can1*Δ deletion strain as highly enriched in the population following canavanine treatment (Figure 1a).

In the robot-assisted experiments, four replicates of a deletant strain for each of the known yeast genes encoding transporter proteins were spotted onto solid medium. Growth on a plate containing canavanine identified only the known canavanine-resistant strain *can1*Δ/*can1*Δ (data not shown), in complete agreement with published data and with our results from the competition experiment described above.

We validated the results from both high-throughput experiments by performing growth experiments in a BioScreen C instrument (Thermo Electron, Helsinki, Finland), which generates robust growth curves under more strictly controlled conditions (Figure 1b). We calculated the maximum growth rate of the WT and *can1*Δ/*can1*Δ strains in the presence of canavanine (0 μM to 100 μM; Figure 1c), and confirmed that, unlike the wild type, *can1*Δ/*can1*Δ mutants are insensitive to canavanine. Furthermore, a competition experiment between canavanine and the native Can1p substrate, arginine, illustrates the fully protective effect of arginine (Figures 1b and 1c). Both of these results suggest that the cellular import of canavanine occurs exclusively via Can1p, as reported previously [23].

### Drugs with a single protein carrier

The two screening procedures identified a number of transporters which clearly represented the sole transporter responsible for the uptake of a particular drug into yeast cells. The first example is similar to that of Can1p transporter and canavanine. We screened for transporters responsible for the uptake of the anticancer drugs 5-fluorocytosine and 5-fluorouracil and, as could have been expected, found that the *fcy2*Δ/*fcy2*Δ mutant was the most resistant strain (Additional Files 1 and 2). Fcy2p is a known cytosine transporter and is so named because of the fluorocytosine-resistant phenotype of its mutant alleles [24].

**Figure 2:**
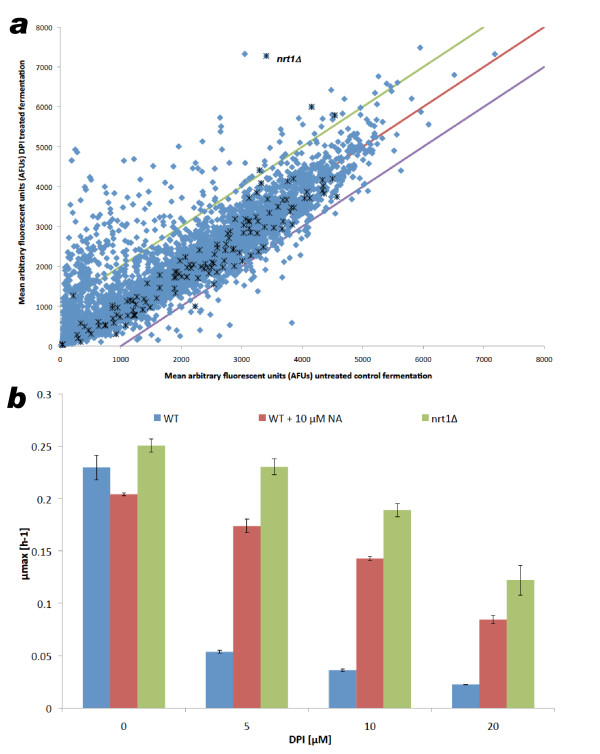
**Competition experiment in the presence of diphenyleneiodonium chloride**. **(a) **Pool competition results for selection for diphenyleneiodonium chloride (DPI) resistance. The abscissa indicates the proportion of each strain in the untreated pool. When treated (y-axis) the ability of the *nrt1*Δ/*nrt1*Δ diploid to resist DPI confers a major growth advantage to the strain such that it outcompetes all others to become the most abundant strain. Green and blue lines (± 1000 y-translation of parity line) detail the boundary within which 98% of deletants are found when comparing untreated controls, so providing a noise estimate. Blue diamonds denote all deletants and black stars identify strains deleted for transporter genes. AFU = the mean arbitrary fluorescent units measured for the TAG4 Arrays of treated (DPI) and untreated (control) competitions between the pools of homozygous deletants. **(b) **Comparison of the maximum specific growth rate achieved by wild type (ydl227cΔ/ydl227cΔ) and *nrt1*Δ/*nrt1*Δ mutant in the presence of 0 to 20 μM DPI and 0 μM or 10 μM of the competitor nicotinic acid. Error bars = standard error of the mean; n = 3. DPI = diphenyliodonium chloride.

**Figure 3:**
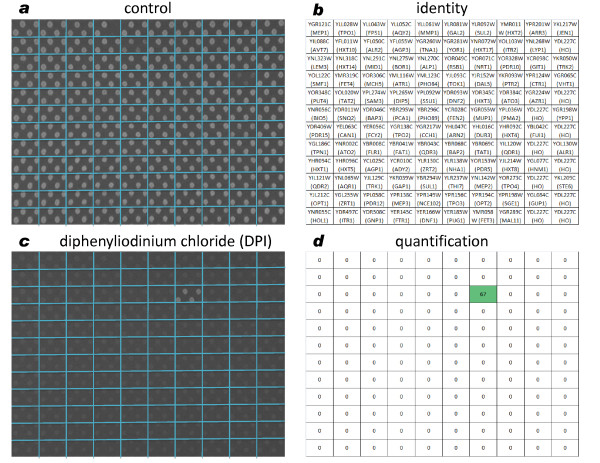
**Identification of a putative **diphenyleneiodonium **chloride transporter in the robot-assisted experiment**. **(a) **Control plate with homozygous deletion mutant strains spotted in quadruplicates (by a Singer RoToR^© ^HAD robot) onto F1 medium agar plate containing 1% dimethyl sulfoxide. **(b) **Identity of deletion mutants spotted onto control and drug plates. **(c) **Homozygous deletion mutant strains spotted in quadruplicates (by a Singer RoToR^© ^HAD robot) onto F1 medium agar plate containing 8 μM diphenyleneiodonium chloride. **(d)**. Quantification of the relative growth between drug and control plates with diphenyleneiodonium chloride resistant strains highlighted in green.

The analysis of data from pool competition experiments with diphenyleneiodonium chloride (DPI), by plotting the mean arbitrary fluorescence of untreated and treated pools, identified the *nrt1*Δ/*nrt1*Δ deletant as highly enriched in the population following DPI treatment (Figure 2a). Robot-assisted experiments using individual transporter deletants spotted onto agar also identified Nrt1p as the most likely DPI transporter (Figure 3). We next performed growth assays on WT and *nrt1*Δ/*nrt1*Δ strains in the presence of increasing DPI concentrations and verified the resistance conferred by the deletion of the candidate transporter (Figure S3a in Additional File 3).

DPI is an inhibitor of reduced nicotinamide adenine dinucleotide phosphate oxidase and related enzymes [25], and bears some structural similarity to nicotinamide riboside. Furthermore, both nicotinamide riboside and thiamine [26] are known to be transported by Nrt1p [27]. Therefore, we investigated whether the native Nrt1p substrates could protect cells against DPI by outcompeting the drug for import via the Nrt1 transporter. Nicotinamide riboside is not commercially available and so the structurally related compounds, nicotinic acid and nicotinamide, were assessed. We found that 10 μM of either nicotinic acid or nicotinamide protects against DPI (at its IC_90_), recovering 80% of the control growth rate (Figure 2b and data not shown). Thiamine, which is imported via Nrt1p with a lower affinity than nicotinamide riboside, was also able to protect cells against DPI, albeit less efficiently than nicotinic acid (Figures S3b and S3c in Additional file 3).

Using robot-assisted experiments, we found that two structurally related antineoplastic drugs, methotrexate and aminopterin, are also potential substrates of the Nrt1 transporter (Additional files 4 and 5). Neither nicotinic acid nor nicotinamide protected against growth-rate inhibition by methotrexate (data not shown). However, final optical density (OD), which broadly equates to biomass yield, can also indicate drug resistance. Using this as the criterion, protection due to 10 μM nicotinamide or nicotinic acid was observed, increasing the final OD from 0.3 without protection to 0.5 with the protective substrate (compared to a final OD of 0.9 for the untreated wild type). At 250 μM, thiamine protects weakly against methotrexate (data not shown).

Robot-assisted experiments also indicated an aminopterin resistance phenotype for the *ctr1Δ/ctr1*Δ (copper transporter) and the *fen2*Δ/*fen2*Δ (pantothenate transporter) mutant strains (Additional file 5). Aminopterin inhibits the activity of dihydrofolate reductase (DHFR), an enzyme that is required for purine biosynthesis and for which there is a high demand in rapidly growing cells [28]. Given that these two deletants have reduced growth rates even in the absence of drugs (data not shown), it may be that the consequent reduced demand for DHFR activity makes them less susceptible to the deleterious effects of DHFR inhibitors such as aminopterin.

Experiments with the alkylating agent iodoacetamide [29] suggested a single transporter, the maltose transporter, Mal11p [30] (Additional file 6); however, this result was only observed on solid medium and not in liquid cultures. Furthermore, this drug/transporter combination seems structurally improbable, and therefore needs to be validated by independent methods to provide a clear picture of whether and how this import operates.

Robot-assisted experiments to identify the transporters of Bay11-7085 (an NF-kappa B inhibitor) [31] and benzbromarone (the uricosuric agent used in the treatment of gout) [32] suggested the uridine permease, Fui1p [33], as the main route for cell entry (Additional Files 7 and 8). However, due to the large number of suppressor mutants of yeast plated on agar containing either of these two drugs, this result could not be validated in liquid cultures. While pool selections performed in the benzbromarone-containing cultures did indicate that the *fui1Δ/fui1*Δ mutant was among the five most enriched deletants in the population, the enrichment measured was below our standard threshold of significance (data not shown).

Mitoxantrone is an antineoplastic agent that acts by inhibiting Type II topoisomerases [34]. In robot-assisted experiments, we found that the most likely route for mitoxantrone to enter yeast cells is via the low-affinity amino-acid permease, Agp1p [35] (Additional File 9). Interestingly, the same route was suggested for protoporphyrin import (Additional file 10). Protoporphyrin is a tetrapyrrole used as a carrier for divalent cations [36] and it has been previously suggested that it is imported into yeast cells via Pug1p [37]. However, in our strain background and experimental conditions (robot-assisted experiments), *pug1*Δ*/pug1*Δ mutants were not phenotypically different from the control strains (Additional file 10).

### Drugs for which multiple transporters were identified

Quantitative analysis of the robot-assisted experiments performed on cisplatin plates identified the purine and cytosine permease, Fcy2p [24], as the main import route for this anticancer drug (Additional File 11). The experiments also identified the phospholipid transporter, Lem3p [38], as a putative cisplatin transporter; however, this was not reproduced in liquid cultures. Interestingly, the arsenite and antimonite transporter, Fps1p, as well as the choline/ethanolamine transporter, Hnm1p, also showed resistance to cisplatin, albeit to a level below our threshold of 3 SD from the plate average (Additional File 12). As with many of the examples in this section, experiments with double (in this case, *fps1*Δ *hnm1*Δ) or multiple mutants might reveal a very strong resistance phenotype and establish the relative contribution of each of the carrier proteins to the transport of the drug.

Tunicamycin is an antibiotic that inhibits protein N-glycosylation [39] and therefore is used experimentally to induce the unfolded protein response [40]. Robot-assisted experiments on tunicamycin plates identified five transporter gene deletions conferring resistance to the drug: *lem3*Δ/*lem3*Δ, *dnf2*Δ/*dnf2*Δ, *pca1*Δ/*pca1*Δ, *pho89*Δ/*pho89*Δ and *qdr2*Δ/*qdr2*Δ (Additional File 13). Both Lem3p and Dnf2p are phospholipid transporters [38,41] and therefore might contribute to tunicamycin import by binding the hydrophobic tail common to all forms of the drug (Additional file 14). Pho89p and Pca1p are phosphate [42] and metal transporters [43], respectively, and therefore are unlikely to be responsible for the direct uptake of the drug. Qdr2p, on the other hand, is a known pleiotropic drug transporter that might well assist in tunicamycin import [44]. More examples of the indirect effect of transporters on drug uptake or efficacy are given below.

### Drugs for which transporters have an indirect effect on their efficacy

Robot-assisted experiments linked the Fcy2p transporter (required for the import of 5-fluorocytosine and 5-fluoruracil, see above) to the import of the antifungal drug, fluconazole (Additional File 15). Deletions of three additional transporter genes (*FET3*, *FTR1*, and *ITR1*) also conferred resistance to fluconazole. Fluconazole acts by inhibiting the cytochrome P450 enzyme 14-α-demethylase [45], one of only three P450s in *S. cerevisiae*. Cytochrome P450s are heme-containing proteins, and Fet3p and Ftr1p are known iron import routes [46,47]. Therefore, it is plausible that the resistance to fluconazole that we observed with *fet3Δ/fet3*Δ and *ftr1Δ/ftr1*Δ mutants might be an influence on the drug's target, rather than its import. Itr1p is a myoinositol transporter and its role in fluconazole import is not clear [48]. Interestingly, robot-assisted experiments also identified Itr1p as the putative transporter of two more azole antifungal drugs, ketoconazole (80 μM) and clotrimazole (32 μM) (Additional Files 16 and 17), which also target the cytochrome P450 family. This result establishes a new link between Itr1p and azoles. At lower clotrimazole concentrations (25 μM), *fet3Δ/fet3*Δ and *ftr1Δ/ftr1*Δ mutants were also resistant to the drug (Additional file 18).

Robot-assisted experiments on cantharidin (a drug used for the topical treatment of warts and other skin tumors) identified no transporter deletion strain that showed resistance to the drug at a significance level above our standard threshold of 3 SD over the plate average (data not shown). However, if we lowered the stringency of the screen to include hits 2.5 SD above the plate average (Additional File 19), we could identify Snq2p, Cch1p, Mid1p, Pho89p or Fen2p as possible uptake routes. Snq2p is a multidrug transporter and therefore a plausible cantharidin import route [49]. Cch1p and Mid1p work together to mediate calcium import [50]. Whilst it is reassuring to identify two proteins that are known to work in tandem, it seems unlikely that calcium channels are directly responsible for cantharidin import. Pho89p is responsible for phosphate uptake and cantharidin is a phosphatase inhibitor [42,51]. Therefore, we might infer that a phosphate imbalance due to the *pho89*Δ/*pho89*Δ mutation might be responsible for the observed resistance and that Pho89p is not directly responsible for cantharidin uptake. The structure of cantharidin does not resemble known Fen2p substrates (tail of two carbons and a carboxyl group; Additional file 20); however, validation assays in liquid cultures verified the resistance to cantharidin observed in *fen2*Δ/*fen2*Δ strains (data not shown).

Robot-assisted experiments with the antimalarial drug, artesunate [52], did not provide strong hits using the significance threshold of 3 SD above the plate average (data not shown). However, when looking for the strains with growth 2 SD above the plate average, we identified *cch1Δ/cch1*Δ [50], *mid1Δ/mid1*Δ [50] and *fen2*Δ/*fen2*Δ as artesunate-resistant strains (Additional File 21). The overlap between the cantharidin and artesunate hits is quite striking; especially considering that cantharidin has also been demonstrated to be an antiparasitic (antileishmanial) agent [53]. After drug-induced cell stress, yeast cells frequently undergo changes in intracellular calcium concentrations mediated by Cch1p-Mid1p [54], therefore the role of *cch1*Δ/*cch1*Δ and *mid1*Δ/*mid1*Δ deletions in resistance to artesunate and cantharidin is unlikely to be due to a direct role in drug import. However, the pantothenate transporter Fen2p is a possible artesunate import route as it bears the carboxyl tail observed in other Fen2p substrates (aminopterin and pantothenate) and in the substrates of related proteins, Vht1p and Dal5p (Additional File 20) [55,56].

The drug 1,10-phenanthroline is a heterocyclic organic compound that forms strong complexes with most metal ions [57]. Interestingly, robot-assisted experiments using this drug suggested the high-affinity copper transporter, Ctr1p [58], as the major route of cellular ingress for phenanthroline (Additional file 22). It remains to be demonstrated if phenanthroline import is aided by the formation of a complex with copper or if the resistance we observed is an indirect effect of an intracellular copper imbalance (we observed that strains lacking the iron transporters Ftr1p [47] or Fet3p [46] are hypersensitive to phenanthroline). Ammonium pyrrolidine dithiocarbamate is a metal chelator that induces G1 cell cycle arrest [59]. Therefore, it was not surprising to identify the strain lacking the cadmium transporter, Pca1p, as the most resistant strain in a robot-assisted experiment (Additional File 23). The 1,10-phenanthroline resistance observed in a *pca1*Δ/*pca1*Δ strain could be due to an indirect effect caused by metal imbalance and not by a direct role of Pca1p in drug import.

### Drugs for which no transporter could be identified

At the concentrations tested (Table 1 and data not shown), we could not identify candidate transporters for 3,4-dichloroisocoumarin, N-phenylanthranilic acid, tamoxifen, tetraethylthiuram disulphide (the alcohol deterrent, disulfiram), vanillylmandelic acid (the tyrosine mono-oxygenase inhibitor, metyrosine) or ZM39923 (the Janus kinase inhibitor). With our current experimental set-up, it is not possible to determine whether this was due to passive diffusion of the drug via the plasma membrane, presence of multiple transporters equally capable of importing the drugs (in which case, only double mutants would show adequate resistance to the drug) or whether the strain deleted for the correct transporter was not present in our collection. Even if no transporter is present in *S. cerevisiae*, the possibility that human cells may contain specific transporters for these drugs cannot be excluded, since bioinformatic analyses predict that the human genome encodes 1022 transporter proteins, compared with yeast's 318 [60].

**Table 1:** Summary of the transporters responsible for the uptake of cytotoxic drugs.

Drug	Pool hits	Robot [μM]	Robot hits	Indirect effect	Verification hits	Competitor	Lipinski's **Rules **[3]
Aminopterin		2	*nrt1*Δ*, fen2*Δ	*ctr1*Δ	*nrt1*Δ, c*tr1*Δ	NT	Fail

Amm. pyrrolidine thiocarbamate		20		*pca1*Δ	NT	NT	Pass

Artesunate		100	*fen2*Δ (2*σ)	*cch1*Δ*, mid1*Δ (2*σ)	NT	NT	Pass

Bay 11-7985		10	*fui1*Δ		NT	NT	Pass

Benzbromarone	None	28	*fui1*Δ		NT	NT	Fail

Canavanine	*can1*Δ	5	*can1*Δ		*can1*Δ	pass	Fail

Cantharidin		30	*fen2*Δ*, snq2*Δ (2.5*σ)	*cch1*Δ*, mid1*Δ*, pho89*Δ (2.5*σ)	*fen2*Δ *pho89*Δ (others NT)	NT	Pass

Cisplatin		50	*fcy2*Δ*, lem3*Δ*, (fat1*Δ*, fps1*Δ*, hnm1*Δ 2*σ)		*fcy2*Δ*, (fat1*Δ*, fps1*Δ*, hnm1*Δ NT)	NT	Pass

Clotrimazole		25	*itr1*Δ	*ftr1*Δ *fet3*Δ	NT	NT	Fail

3,4-Dichloroisocoumarin		8	None				Pass

Diphenyleneiodonium chloride	*nrt1*Δ	8	*nrt1*		*nrt1*Δ	pass	Pass

Fluconazole		100	*itr1*Δ *fcy2*Δ	*ftr1*Δ *fet3*Δ	NT	NT	Pass

5-Fluorocytosine		158	*fcy2*		*fcy2*Δ	NT	Pass

5-Fluorouracil		158	*fcy2*Δ	*fen2*Δ	*fcy2*Δ *fen2*	NT	Pass

Iodoacetamide		20	*mal11*Δ (2.5*σ)		Reproducible only on solid	NT	Pass

Ketoconazole		80	*itr1*Δ *fat1*Δ		NT	NT	Fail

Methotrexate		100	*nrt1*Δ		*nrt1*Δ	pass	Fail

Mitoxantrone		75	*agp1*		NT	NT	Fail

1,10-Phenanthroline		14		*ctr1*Δ	NT	NT	Pass

N-Phenylanthranilic Acid		100	None				Pass

Protoporphyrin		600	*agp1*Δ		NT	NT	Fail

Tamoxifen		730	None				Fail

Tetraethylthiuram disulfide		10		*pdr5*Δ (2*σ)	NT	NT	Fail

Tunicamycin		4	*lem3*Δ *dnf2*Δ *qdr2*Δ	*pca1*Δ *pho89*Δ	all confirmed	NT	Fail

Vanillylmandelic acid		647	None				Pass

ZM 39923		14	None				Fail

NT indicates liquid verification or competitor experiments were not tested.

## Discussion

The importance of carriers in drug uptake has, until recently, been much overlooked in favor of the idea of drug uptake by diffusion through the lipid bilayer, despite persuasive arguments and extensive evidence to the contrary [60-65]. Carriers are an important component of cellular biochemistry (and of biotechnological production [66]), with many hundreds known in both yeast [67] and human [68,69] cells. To assess which drugs use which transporters, we have employed two high-throughput experimental platforms to identify new drug-transporter interactions. Through these targeted validation experiments, including protection with known substrates, we have been able to identify and/or confirm the transporters required for uptake of 18 of 26 drugs tested (Table 1).

The approach we have described relies on substrates being cytotoxic, and upon the identification of the optimum drug concentration for each screen. Furthermore, due to the fact that our method is based on the use of single deletion mutants, we would not always be able to detect redundant transporters. Therefore, a collection with double transport mutants would provide invaluable information about possible transporters for those drugs not yielding hits.

For hundreds of transporters to mediate the uptake of tens of thousands of diverse compounds, as must occur if transporters dominate uptake, considerable transporter promiscuity is required. Certain solute carriers present in mammalian genomes are known to transport extremely diverse substrates, with PepT1 being a particularly clear example for which early structure-activity relationships have been defined [70]. Yet such structural insights are exceptional and, generally, the chemical basis of promiscuous transporter function is not well understood. Here, we have identified several examples of yeast transporters with multiple and diverse substrates. Fen2p has been shown to mediate the uptake of artesunate, pantothenate and aminopterin, which bear the characteristic carboxyl group of other Fen2p substrates and the substrates of related transporters (Dal5p: allantoate, ureidosuccinate; Vht1p: biotin), but are otherwise structurally dissimilar. Our experiments also link 5-fluorouracil and cantharidin to Fen2p, which do not bear the carboxyl group. This suggests Fen2p might transport an even broader range of substrates, although we cannot eliminate the possibility that the gene deletion confers resistance indirectly (as indeed is the case for fenpropimorph, after which the protein is named).

The experimental survey generated many such links between drugs and transporters that are difficult to rationalize, as well as links with tantalizing (but far from conclusive) structural similarities - such as that between benzbromarone and the uridine substrate of Fui1p. This lack of a substrate-level understanding of transporter function particularly highlights the need for methods such as those developed here, which are capable of uncovering links one would not otherwise anticipate. It seems clear, however, that, in combination, a set of transporters are indeed capable of the promiscuity necessary to mediate the uptake of very diverse substrates.

Drug development is a multi-objective optimization task, with major components of the objective function being terms describing the pharmacokinetic processes of drug absorption, tissue distribution and excretion, all of which involve uptake across cellular membranes. To understand pharmacokinetics properly and mechanistically, therefore, requires knowledge of the interactions between transporters (including genetic variants) and their substrates (primarily drugs, nutrients, and endogenous metabolites). Allelic variation data based on the knowledge of these carriers will feed into structure-activity relationship modeling to allow the prediction of likely substrates from large drug libraries [21], and into integrative systems biology models [71] using a patient's individual genotype to move towards delivering personalized medicine.

## Conclusions

This work has exploited the gene-deletion collection of the model eukaryote, *S. cerevisiae*, to employ two chemical genomics platforms with which to identify drug carriers responsible for the uptake of a range of very diverse compounds. The first involves competition between deletion mutants in liquid culture, while the second uses a robot to seed arrays of mutants on agar - in both systems the impact of drugs on the mutants' growth may readily be measured and the genes specifying drug carriers identified by the drug resistance of their cognate deletion mutants. In this way, we have provisionally identified the protein carriers mediating the entry, into yeast, of 18 of 26 drugs studied. Moreover, the impact of the deletion mutants on drug entry firmly establishes that transport via protein carriers, rather than simple diffusion, is likely to be the main route of cellular ingress for many drugs.

## Methods

### Strains and culture conditions

The homozygous deletion pool and individual homozygous and heterozygous deletion strains used were generated in the *S. cerevisiae *deletion project [16]. The parental strain Y23935 (*MAT**a**/α his3Δ1/his3Δ1 leu2Δ0/leu2Δ0 lys2Δ0/LYS2 MET15/met15Δ0 ura3Δ0/ura3Δ0 *ydl227c::*kan*MX4/YDL227c) was used as the WT control throughout (referred to as the WT or standard strain). YDL227c is the open reading-frame of the *HO *gene, which is not expressed in diploid cells; its deletion has no measurable effect on growth rate. Strains were routinely grown in F1 minimal medium [45,46] containing 0.5% glucose as the carbon source or 2% glucose for the robot-generated arrays (Additional file 24). Strains were maintained on 2% yeast extract peptone dextrose (YPD) agar, supplemented with 200 mg/L of Geneticin (G-418; Sigma, Sigma-Aldrich, Gilligham, Dorset, UK). Minimal medium without non-essential amino acids was used throughout growth experiments to minimize possible competition of drug uptake with natural substrates.

### BIOSCREEN experiments and drug screening

Strains were grown on a BIOSCREEN C (distributed by Thermo Electron) in triplicate in a total assay volume of 300 μL at 30°C with intensive on/off shaking [47]. The OD was measured at 600 nm every 10 minutes. For initial screening to determine cytotoxicity, strains were challenged with drugs from the Library of Pharmacologically Active Compounds (LOPAC; Sigma-Aldrich, Dorset, UK) and National Cancer Institute (NCI) Mechanistic and Diversity sets http://dtp.nci.nih.gov/index.html. The maximum final concentration of a dimethyl sulfoxide (DMSO) solution of a drug tested was 100 μM for LOPAC and NCI Diversity sets, and 10 μM for the NCI Mechanistic set, since final DMSO concentrations of > 1% (v/v) reduced the maximum specific growth rate of *S. cerevisiae *significantly (data not shown).

### Pool experiments

Pool experiments were performed as 1.2-liter batch fermentations using the homozygous deletant pool. The deletant pool was stored at -80°C and 200 μL was inoculated into 50 mL F1 medium (0.5% glucose) and incubated on a rotary shaker at 30°C overnight (12 hours). The overnight culture was harvested by centrifugation and the pellet resuspended in 50 mL of fresh F1 medium. Twelve milliliters (1% final concentration) of inoculum were injected into a 1200 mL aerated (1 gas volume flow per unit of liquid volume per minute (vvm)), stirred (700 rpm) and heated (30°C) Applikon 1030 bioreactor (FT Applikon Ltd., Tewkesbury, UK) with a 2.3 L full working volume. The pH was kept constant at 4.5 using 1M potassium hydroxide.

Prior to inoculation, drug solutions were added to the fermenter at their IC_90 _concentrations. In the control, untreated cells grew under equivalent culture conditions. For this type of experiment, overall runtime is not a comparative measure because the strains in the population each respond differently to drug treatment. Accordingly, the final sample was taken when the OD measurement indicated the transition from the exponential growth phase to deceleration phase, which coincides with a sharp drop in carbon dioxide (CO_2_) evolution. Growth and the end-point of growth were monitored using OD_600 _and CO_2 _evolution, measured online via an external CO_2 _gas analyzer (Tandem Gas analyzer, Magellan Instruments, Lipenhoe, UK). Cells were harvested by centrifugation and the pellets stored at -80°C prior to analysis.

### DNA extraction, TAG4 array hybridization

DNA extraction, polymerase chain reactions, TAG4 array hybridization and data analysis were performed according to the methods of Pierce and co-workers [72] using their normalization protocol.

### Preparation of the master-plates for the robot-assisted experiments

Yeast strains with deletions of 111 genes encoding plasma membrane transporters http://http:\\www.yeastgenome.org, as well as multiple copies of the WT control strain [KanMX deletion cassette in the *HO* (YDL227c) locus] were inoculated into 70 μL of YPD in duplicate in a 384-well plate (master-plate). Where possible the homozygous mutants were used, but in the case of the essential genes *VHT1 *(YGR065c), *YPP1 *(YGR198w) and *ALR1 *(YOL130w); or when homozygous deletion strains were not available in our strain collection [*STE6 *(YKL209c)], the corresponding heterozygous strain was used instead. To minimize problems with edge effect, we placed WT strains on all the border wells of our master plates. The master-plate was incubated at 30°C for 36 hours to ensure that each strain had grown to the stationary phase, in order to homogenize the growth throughout the plate.

### Drug selection and preparation of the test-plates

We selected 26 compounds cytotoxic in yeast, 14 of which pass the Lipinski's rule of five (all compounds were purchased from Sigma). Stock solutions of each drug were prepared in water, ethanol or DMSO (according to the compound solubility). Adequate volumes of these stock solutions were added to 40 mL of F1 minimal media to make plates with the final drug concentrations indicated in Table 1. For the stock solutions in DMSO, the concentration of the solvent in the final plate was never greater than 1% by volume, since high DMSO concentrations affect yeast growth (data not shown). The cultures in the 384-well master-plate were spotted in duplicate onto the plates using a Singer RoToR^© ^HAD (Singer Instrument Co., Watchet, Somerset, UK) robot to generate a test plate with 768 spots, that is, each mutant in quadruplicate. The cells were allowed to grow for at least 48 hours at 30°C, at which point images of the plates were captured on the standard gel documentation system, Gel Doc 2000 (Bio-Rad, Bio-Rad UK Ltd, Hemel Hempstead, UK), and saved as JPEG images.

### Quantification of growth on robot generated plates

The quantification of yeast growth on robot-generated plates was based on the method described in Bilsland *et al*. [73], with a few modifications to account for the number of colonies on each plate. MATLAB was used to convert the JPEG images to three-dimensional intensity matrices, and the intensities from the blue channel were used to quantify the colony sizes. The corners of the plate were identified manually and, accordingly, a 'window size' was calculated as the larger of the following two values: the width of the image divided by the number of columns or length of the image divided by the number of rows. The image was then partitioned into equal-sized diamond-shaped windows, with diagonals the same length as the 'window size' calculated previously, and each window framing a colony. The pixels with intensity 25% higher than the minimum intensity of the colony window were counted and the total count was assigned as the size of the colony.

The colonies on the edges of the plates, the WT buffer, were excluded from further analysis as the sizes of these colonies are biased by 'edge effects' (the decreased competition resulting from being on the edge). For the four spots corresponding to each particular mutant, the median size was calculated. Because the strains did not all grow at the same rate on control plates, this median value was then divided by the median value of the four spots of the corresponding mutant on the relevant control plate. Finally, this value was multiplied by 100. Strains with sizes more than 2.5 SD below the plate average were highlighted red, signifying sensitivity, and strains with sizes more than 3 SD above the plate average were highlighted green, signifying resistance. In some cases, the threshold for resistance was lowered to 2.5 or 2 SD, in order to compensate for the effects of extreme outliers on the average value.

## Authors' contributions

DBK and SGO conceived the project and the initial experimental design. PD selected the drugs and analyzed drug/substrate similarities. All pool competition experiments were performed by KL, who piloted the robot-assisted experiments together with EB. All data presented on robot-assisted screens was generated by EB and HJM, and analyzed using a protocol developed by PP. All authors participated in the writing and revision of the manuscript, and read and approved the final version.

## Supplementary Material

Additional file 1 **Identification of a putative 5-fluorocytosine transporter by the robot-assisted experiment**. **(a) **Control plate with homozygous deletion mutant strains spotted in quadruplicates (by a Singer RoToR^© ^HAD robot) onto F1 medium agar plate containing 1% DMSO. **(b) **Identity of deletion mutants spotted onto control and drug plates. **(c) **Homozygous deletion mutant strains spotted in quadruplicates (by a Singer RoToR^© ^HAD robot) onto F1 medium agar plate containing 158 μM 5-fluorocytosine. **(d) **Quantification of the relative growth between drug and control plates with 5-fluorocytosine resistant strains highlighted in green. Click here for file

Additional file 2 **Identification of a putative 5-fluorouracil transporter by the robot-assisted experiment**. **(a) **Control plate with homozygous deletion mutant strains spotted in quadruplicates (by a Singer RoToR^© ^HAD robot) onto F1 agar medium containing 1% DMSO. **(b) **Identity of deletion mutants spotted onto control and drug plates. **(c)**. Homozygous deletion mutant strains spotted in quadruplicates (by a Singer RoToR^© ^HAD robot) onto F1 medium agar plate containing 158 μM 5-fluorouracil. **(d) **Quantification of the relative growth between drug and control plates with 5-fluorouracil resistant strains highlighted in green. Click here for file

Additional file 3 **Validation of Nrt1p as the main DPI transporter**. **(a) **Growth curves of wild type (ydl227cΔ/ydl227cΔ) and *nrt1Δ/nrt1*Δ yeast strains in the presence of various DPI concentrations. **(b) **Comparison of the maximum specific growth rate achieved by the WT strain in the presence of various concentrations of DPI and the competitor nicotinic acid. **(c) **Comparison of the maximum specific growth rate achieved by the WT strain in the presence of various concentrations of DPI and the competitor thiamine. Error bars = standard error of the mean; n = 3. Click here for file

Additional file 4 **Identification of putative methotrexate transporters by the robot-assisted experiment**. **(a) **Control plate with homozygous deletion mutant strains spotted in quadruplicates (by a Singer RoToR^© ^HAD robot) onto F1 medium agar plate containing 1% DMSO. **(b) **Identity of deletion mutants spotted onto control and drug plates. **(c) **Homozygous deletion mutant strains spotted in quadruplicates (by a Singer RoToR^© ^HAD robot) onto F1 medium agar plate containing 100 μM methotrexate. **(d) **Quantification of the relative growth between drug and control plates with methotrexate resistant strains highlighted in green. Click here for file

Additional file 5 **Identification of putative aminopterin transporters by the robot-assisted experiment**. **(a) **Control plate with homozygous deletion mutant strains spotted in quadruplicates (by a Singer RoToR^© ^HAD robot) onto F1 medium agar plate containing 1% DMSO. **(b) **Identity of deletion mutants spotted onto control and drug plates. **(c) **Homozygous deletion mutant strains spotted in quadruplicates (by a Singer RoToR^© ^HAD robot) onto F1 medium agar plate containing 2 μM aminopterin. **(d) **Quantification of the relative growth between drug and control plates with aminopterin resistant strains highlighted in green. Click here for file

Additional file 6 **Identification of a putative iodoacetamide transporter by the robot-assisted experiment**. **(a) **Control plate with homozygous deletion mutant strains spotted in quadruplicates (by a Singer RoToR^© ^HAD robot) onto F1 medium agar plate containing 1% DMSO. **(b) **Identity of deletion mutants spotted onto control and drug plates. **(c) **Homozygous deletion mutant strains spotted in quadruplicates (by a Singer RoToR^© ^HAD robot) onto F1 medium agar plate containing 20 μM iodoacetamide. **(d) **Quantification of the relative growth between drug and control plates with iodoacetamide resistant strains highlighted in green. Click here for file

Additional file 7 **Identification of putative Bay11-7085 transporters by the robot-assisted experiment**. **(a) **Control plate with homozygous deletion mutant strains spotted in quadruplicates (by a Singer RoToR^© ^HAD robot) onto F1 medium agar plate containing 1% DMSO. **(b) **Identity of deletion mutants spotted onto control and drug plates. **(c) **Homozygous deletion mutant strains spotted in quadruplicates (by a Singer RoToR^© ^HAD robot) onto F1 medium agar plate containing 10 μM Bay11-7085. **(d) **Quantification of the relative growth between drug and control plates with Bay11-7085 resistant strains highlighted in green. Click here for file

Additional file 8 **Identification of putative benzbromarone transporters by the robot-assisted experiment**. **(a) **Control plate with homozygous deletion mutant strains spotted in quadruplicates (by a Singer RoToR^© ^HAD robot) onto F1 medium agar plate containing 1% DMSO. **(b) **Identity of deletion mutants spotted onto control and drug plates. **(c) **Homozygous deletion mutant strains spotted in quadruplicates (by a Singer RoToR^© ^HAD robot) onto F1 medium agar plate containing 2 μM benzbromarone. **(d) **Quantification of the relative growth between drug and control plates with benzbromarone resistant strains highlighted in green. Click here for file

Additional file 9 **Identification of putative mitoxantrone transporters by the robot-assisted experiment**. **(a) **Control plate with homozygous deletion mutant strains spotted in quadruplicates (by a Singer RoToR^© ^HAD robot) onto F1 medium agar plate containing 1% DMSO. **(b) **Identity of deletion mutants spotted onto control and drug plates. **(c) **Homozygous deletion mutant strains spotted in quadruplicates (by a Singer RoToR^© ^HAD robot) onto F1 medium agar plate containing 75 μM mitoxantrone. **(d) **Quantification of the relative growth between drug and control plates with mitoxantrone resistant strains highlighted in green. Click here for file

Additional file 10 **Identification of putative protoporphyrin transporters by the robot-assisted experiment**. **(a) **Control plate with homozygous deletion mutant strains spotted in quadruplicates (by a Singer RoToR^© ^HAD robot) onto F1 medium agar plate containing 1% DMSO. **(b) **Identity of deletion mutants spotted onto control and drug plates. **(c) **Homozygous deletion mutant strains spotted in quadruplicates (by a Singer RoToR^© ^HAD robot) onto F1 medium agar plate containing 600 μM protoporphyrin. **(d) **Quantification of the relative growth between drug and control plates with protoporphyrin resistant strains highlighted in green. Click here for file

Additional file 11 **Identification of putative cisplatin transporters by the robot-assisted experiment**. **(a) **Control plate with homozygous deletion mutant strains spotted in quadruplicates (by a Singer RoToR^© ^HAD robot) onto F1 medium agar plate. **(b) **Identity of deletion mutants spotted onto control and drug plates. **(c) **Homozygous deletion mutant strains spotted in quadruplicates (by a Singer RoToR^© ^HAD robot) onto F1 medium agar plate containing 50 μM cisplatin. **(d) **Quantification of the relative growth between drug and control plates with cisplatin resistant strains highlighted in green (3 SD above the plate average). Click here for file

Additional file 12 **Identification of putative cisplatin transporters by the robot-assisted experiment**. **(a) **Control plate with homozygous deletion mutant strains spotted in quadruplicates (by a Singer RoToR^© ^HAD robot) onto F1 medium agar plate containing 1% DMSO. **(b) **Identity of deletion mutants spotted onto control and drug plates. **(c) **Homozygous deletion mutant strains spotted in quadruplicates (by a Singer RoToR^© ^HAD robot) onto F1 medium agar plate containing 50 μM cisplatin. **(d) **Quantification of the relative growth between drug and control plates with cisplatin resistant strains highlighted in green (2 SD above the plate average). Click here for file

Additional file 13 **Identification of putative tunicamycin transporters by the robot-assisted experiment**. **(a) **Control plate with homozygous deletion mutant strains spotted in quadruplicates (by a Singer RoToR^© ^HAD robot) onto F1 medium agar plate containing 1% DMSO. **(b) **Identity of deletion mutants spotted onto control and drug plates. **(c) **Homozygous deletion mutant strains spotted in quadruplicates (by a Singer RoToR^© ^HAD robot) onto F1 medium agar plate containing 4 μM tunicamycin. **(d) **Quantification of the relative growth between drug and control plates with tunicamycin resistant strains highlighted in green. Click here for file

Additional file 14 **Chemical structure of tunicamycin linked to the proposed transporters**. Click here for file

Additional file 15 **Identification of putative fluconazole transporters by the robot-assisted experiment**. **(a) **Control plate with homozygous deletion mutant strains spotted in quadruplicates (by a Singer RoToR^© ^HAD robot) onto F1 medium agar plate containing 1% DMSO. **(b) **Identity of deletion mutants spotted onto control and drug plates. **(c) **Homozygous deletion mutant strains spotted in quadruplicates (by a Singer RoToR^© ^HAD robot) onto F1 medium agar plate containing 100 μM fluconazole. **(d) **Quantification of the relative growth between drug and control plates with fluconazole resistant strains highlighted in green. Click here for file

Additional file 16 **Identification of putative ketoconazole transporters by the robot-assisted experiment**. **(a) **Control plate with homozygous deletion mutant strains spotted in quadruplicates (by a Singer RoToR^© ^HAD robot) onto F1 medium agar plate containing 1% DMSO. **(b) **Identity of deletion mutants spotted onto control and drug plates. **(c) **Homozygous deletion mutant strains spotted in quadruplicates (by a Singer RoToR^© ^HAD robot) onto F1 medium agar plate containing 80 μM ketoconazole. **(d) **Quantification of the relative growth between drug and control plates with ketoconazole resistant strains highlighted in green. Click here for file

Additional file 17 **Identification of putative clotrimazole transporters by the robot-assisted experiment**. **(a) **Control plate with homozygous deletion mutant strains spotted in quadruplicates (by a Singer RoToR^© ^HAD robot) onto F1 medium agar plate containing 1% DMSO. **(b) **Identity of deletion mutants spotted onto control and drug plates. **(c) **Homozygous deletion mutant strains spotted in quadruplicates (by a Singer RoToR^© ^HAD robot) onto F1 medium agar plate containing 32 μM clotrimazole. **(d) **Quantification of the relative growth between drug and control plates with clotrimazole resistant strains highlighted in green. Click here for file

Additional file 18 **Identification of putative clotrimazole transporters by the robot-assisted experiment. (a) **Control plate with homozygous deletion mutant strains spotted in quadruplicates (by a Singer RoToR^© ^HAD robot) onto F1 medium agar plate containing 1% DMSO. **(b) **Identity of deletion mutants spotted onto control and drug plates. **(c) **Homozygous deletion mutant strains spotted in quadruplicates (by a Singer RoToR^© ^HAD robot) onto F1 medium agar plate containing 25 μM clotrimazole. **(d) **Quantification of the relative growth between drug and control plates with clotrimazole resistant strains highlighted in green. Click here for file

Additional file 19 **Identification of putative cantharidin transporters by the robot-assisted experiment**. **(a) **Control plate with homozygous deletion mutant strains spotted in quadruplicates (by a Singer RoToR^© ^HAD robot) onto F1 medium agar plate containing 1% DMSO. **(b) **Identity of deletion mutants spotted onto control and drug plates. **(c) **Homozygous deletion mutant strains spotted in quadruplicates (by a Singer RoToR^© ^HAD robot) onto F1 medium agar plate containing 30 μM cantharidin. **(d) **Quantification of the relative growth between drug and control plates with cantharidin resistant strains highlighted in green (2.5 standard deviations above the plate average). Click here for file

Additional file 20 **Chemical structures of cantharidin, artesunate and aminopterin as well as that of the native substrate carried by the proposed transporter, Fen2p**. Click here for file

Additional file 21 **Identification of putative artesunate transporters by the robot-assisted experiment**. **(a) **Control plate with homozygous deletion mutant strains spotted in quadruplicates (by a Singer RoToR^© ^HAD robot) onto F1 medium agar plate containing 1% DMSO. **(b**. Identity of deletion mutants spotted onto control and drug plates. **(c) **Homozygous deletion mutant strains spotted in quadruplicates (by a Singer RoToR^© ^HAD robot) onto F1 medium agar plate containing 100 μM artesunate. **(d) **Quantification of the relative growth between drug and control plates with artesunate resistant strains highlighted in green (2 SD above the plate average). Click here for file

Additional file 22 **Identification of putative 1,10-phenanthroline transporters by the robot-assisted experiment**. **(a) **Control plate with homozygous deletion mutant strains spotted in quadruplicates (by a Singer RoToR^© ^HAD robot) onto F1 medium agar plate containing 1% DMSO. **(b) **Identity of deletion mutants spotted onto control and drug plates. **(c) **Homozygous deletion mutant strains spotted in quadruplicates (by a Singer RoToR^© ^HAD robot) onto F1 medium agar plate containing 14 μM 1,10-phenanthroline. **(d) **Quantification of the relative growth between drug and control plates with 1,10-phenanthroline resistant strains highlighted in green. Click here for file

Additional file 23 **Identification of putative ammonium pyrrolidine dithiocarbamate transporters by the robot-assisted experiment**. **(a) **Control plate with homozygous deletion mutant strains spotted in quadruplicates (by a Singer RoToR^© ^HAD robot) onto F1 medium agar plate containing 1% DMSO. **(b) **Identity of deletion mutants spotted onto control and drug plates. **(c) **Homozygous deletion mutant strains spotted in quadruplicates (by a Singer RoToR^© ^HAD robot) onto F1 medium agar plate containing 20 μM ammonium pyrrolidine dithiocarbamate. **(d) **Quantification of the relative growth between drug and control plates with ammonium pyrrolidine dithiocarbamate resistant strains highlighted in green. Click here for file

Additional file 24 **F1 minimal medium**. Components I, II and III can be made up together at 5× final concentration and autoclaved. Component III can be made up at 5× final concentration and autoclaved. Component IV (vitamin solution) is filter-sterilized and kept at -20°C; aliquots are added to fresh 1× solution. Component V is made up as 40% w/v stock solution and autoclaved. Click here for file
