# Supplementary figures and images for: Genome-wide assessment of the carriers involved in the cellular uptake of drugs: a model system in yeast
Source: BMC Biol. 2011 Oct 24;9:70. doi: 10.1186/1741-7007-9-70 (PMC3280192; doi:10.1186/1741-7007-9-70)

## Slide 1
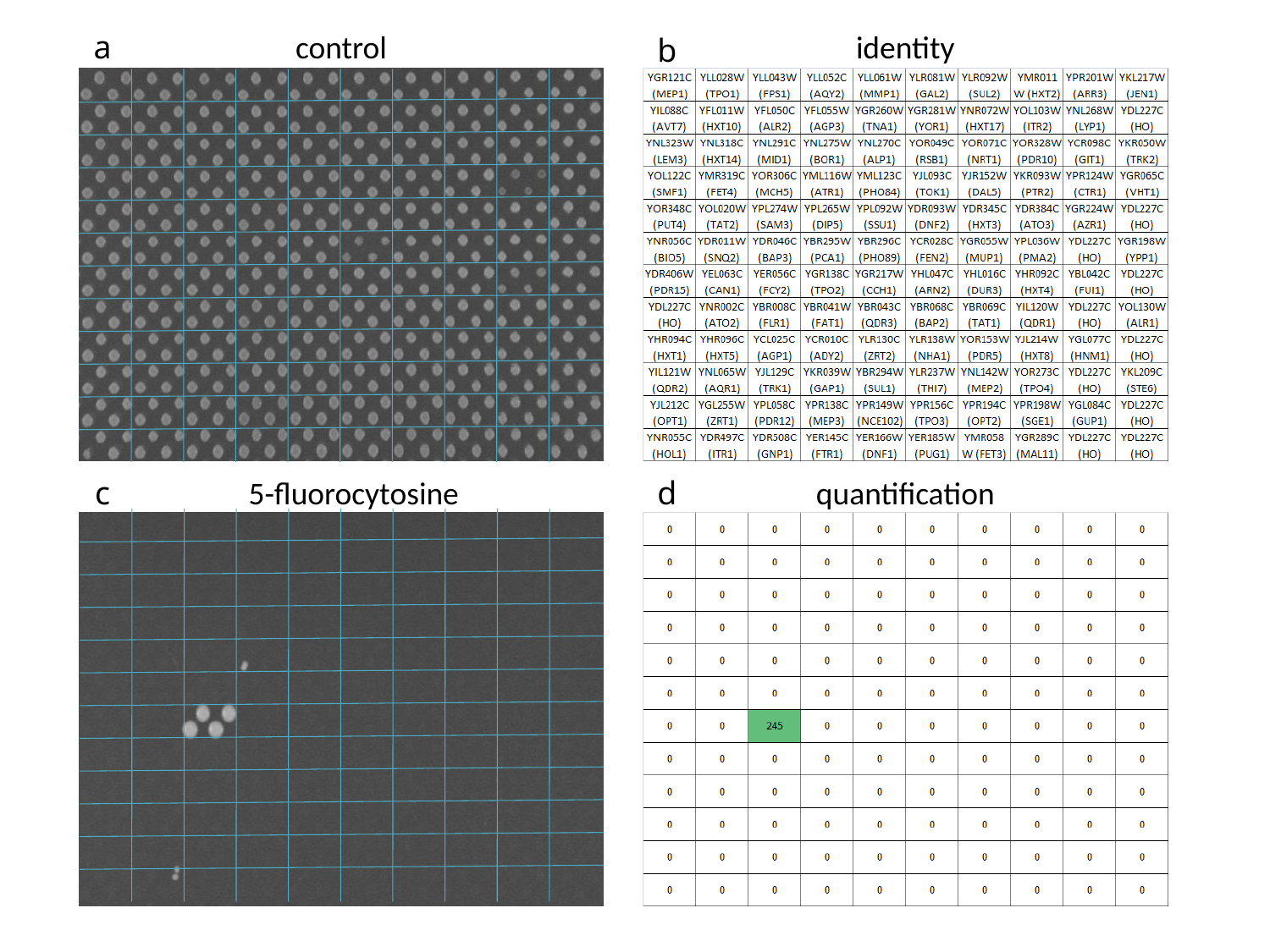

a
control
identity
b
c
5-fluorocytosine
d
quantification

Supplement: Additional file 1 — Identification of a putative 5-fluorocytosine transporter by the robot-assisted experiment. (a) Control plate with homozygous deletion mutant strains spotted in quadruplicates (by a Singer RoToR© HAD robot) onto F1 medium agar plate containing 1% DMSO. (b) Identity of deletion mutants spotted onto control and drug plates. (c) Homozygous deletion mutant strains spotted in quadruplicates (by a Singer RoToR© HAD robot) onto F1 medium agar plate containing 158 μM 5-fluorocytosine. (d) Quantification of the relative growth between drug and control plates with 5-fluorocytosine resistant strains highlighted in green. [file 1741-7007-9-70-S1.PPT]

## Slide 1
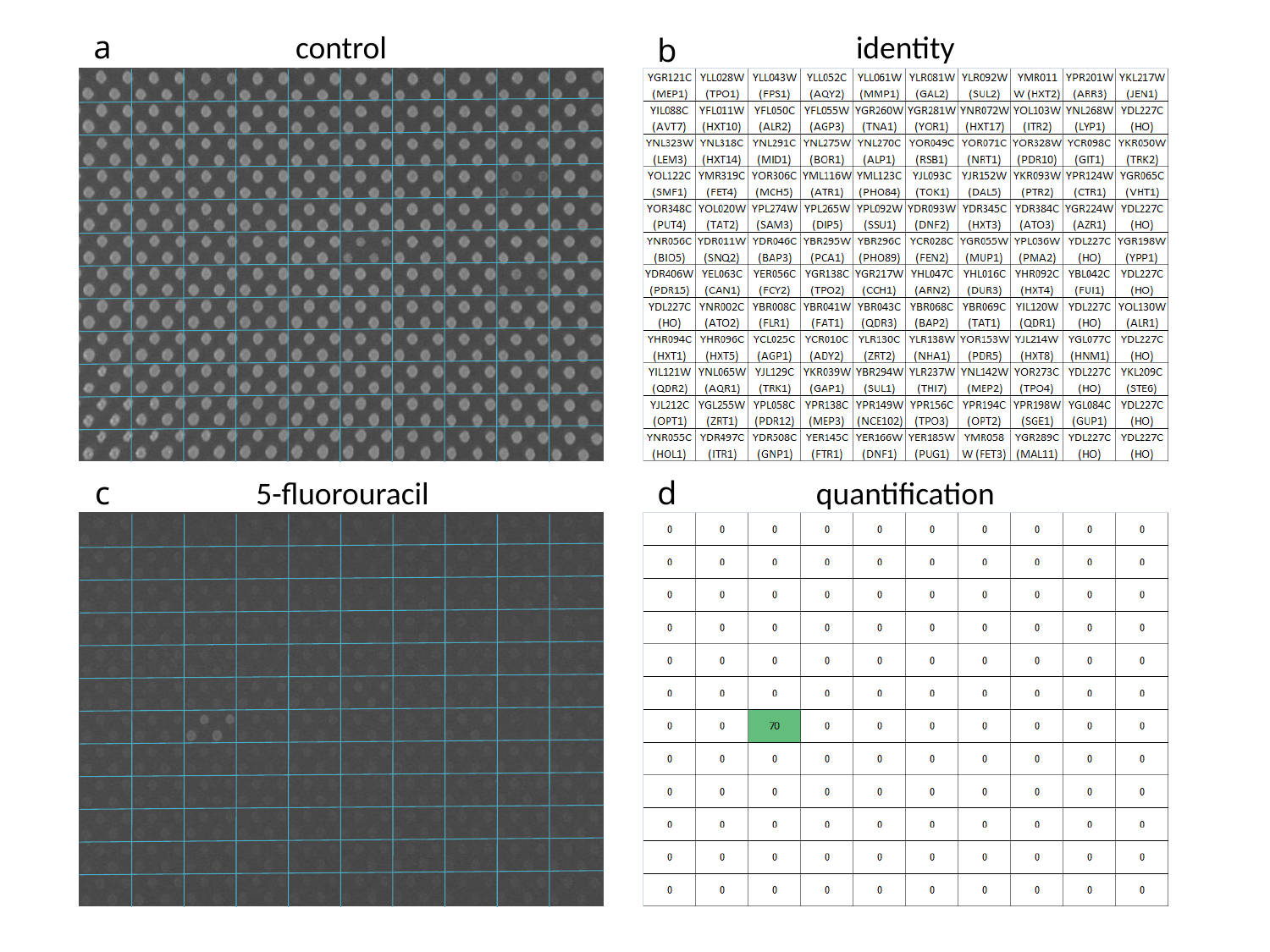

a
control
identity
b
c
5-fluorouracil
d
quantification

Supplement: Additional file 2 — Identification of a putative 5-fluorouracil transporter by the robot-assisted experiment. (a) Control plate with homozygous deletion mutant strains spotted in quadruplicates (by a Singer RoToR© HAD robot) onto F1 agar medium containing 1% DMSO. (b) Identity of deletion mutants spotted onto control and drug plates. (c). Homozygous deletion mutant strains spotted in quadruplicates (by a Singer RoToR© HAD robot) onto F1 medium agar plate containing 158 μM 5-fluorouracil. (d) Quantification of the relative growth between drug and control plates with 5-fluorouracil resistant strains highlighted in green. [file 1741-7007-9-70-S2.PPT]

## Slide 1
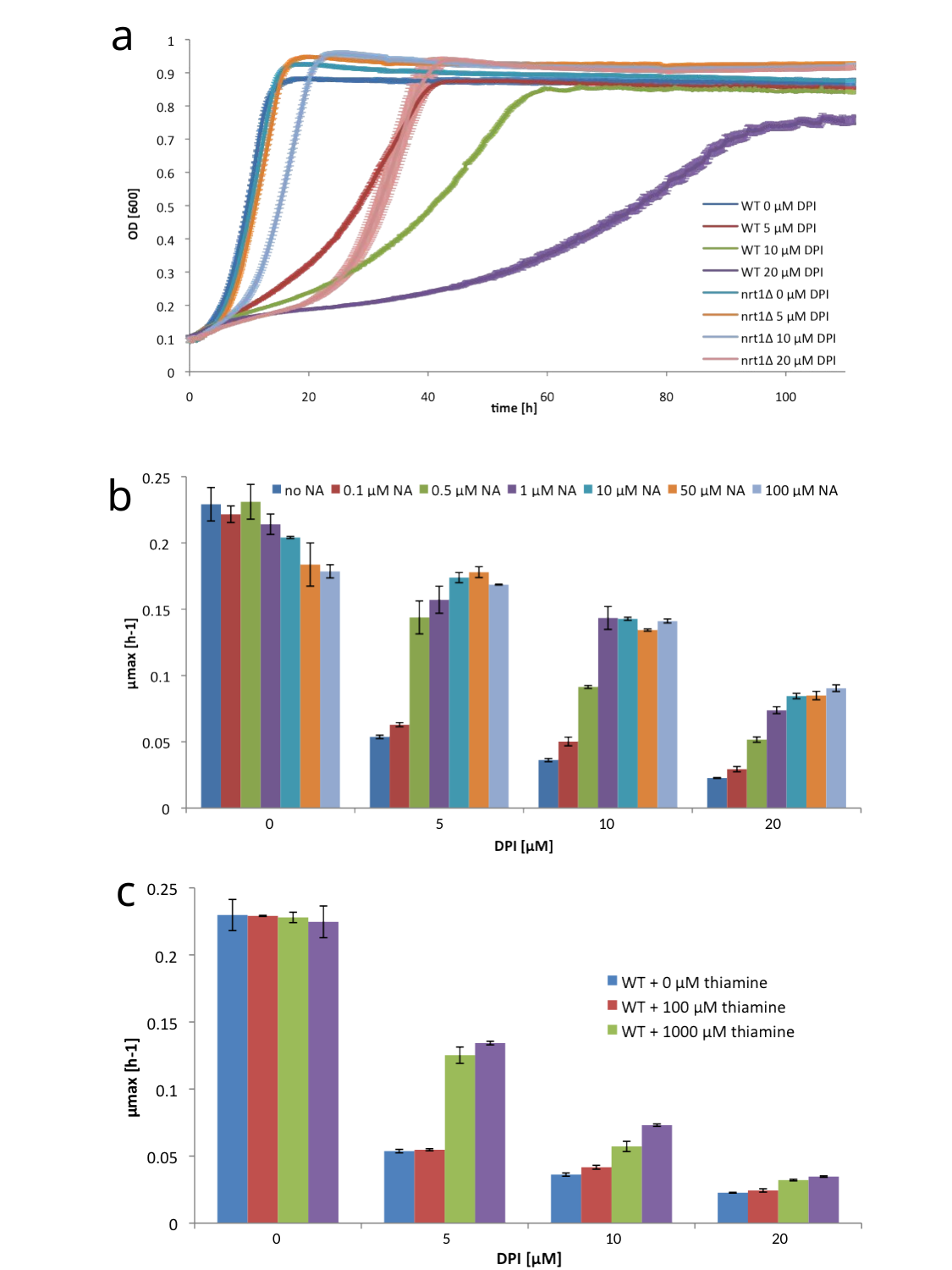

a
b
0		 5	 	 10	 20
c
0		 5	 	 10	 20

Supplement: Additional file 3 — Validation of Nrt1p as the main DPI transporter. (a) Growth curves of wild type (ydl227cΔ/ydl227cΔ) and nrt1Δ/nrt1Δ yeast strains in the presence of various DPI concentrations. (b) Comparison of the maximum specific growth rate achieved by the WT strain in the presence of various concentrations of DPI and the competitor nicotinic acid. (c) Comparison of the maximum specific growth rate achieved by the WT strain in the presence of various concentrations of DPI and the competitor thiamine. Error bars = standard error of the mean; n = 3. [file 1741-7007-9-70-S3.PPT]

## Slide 1
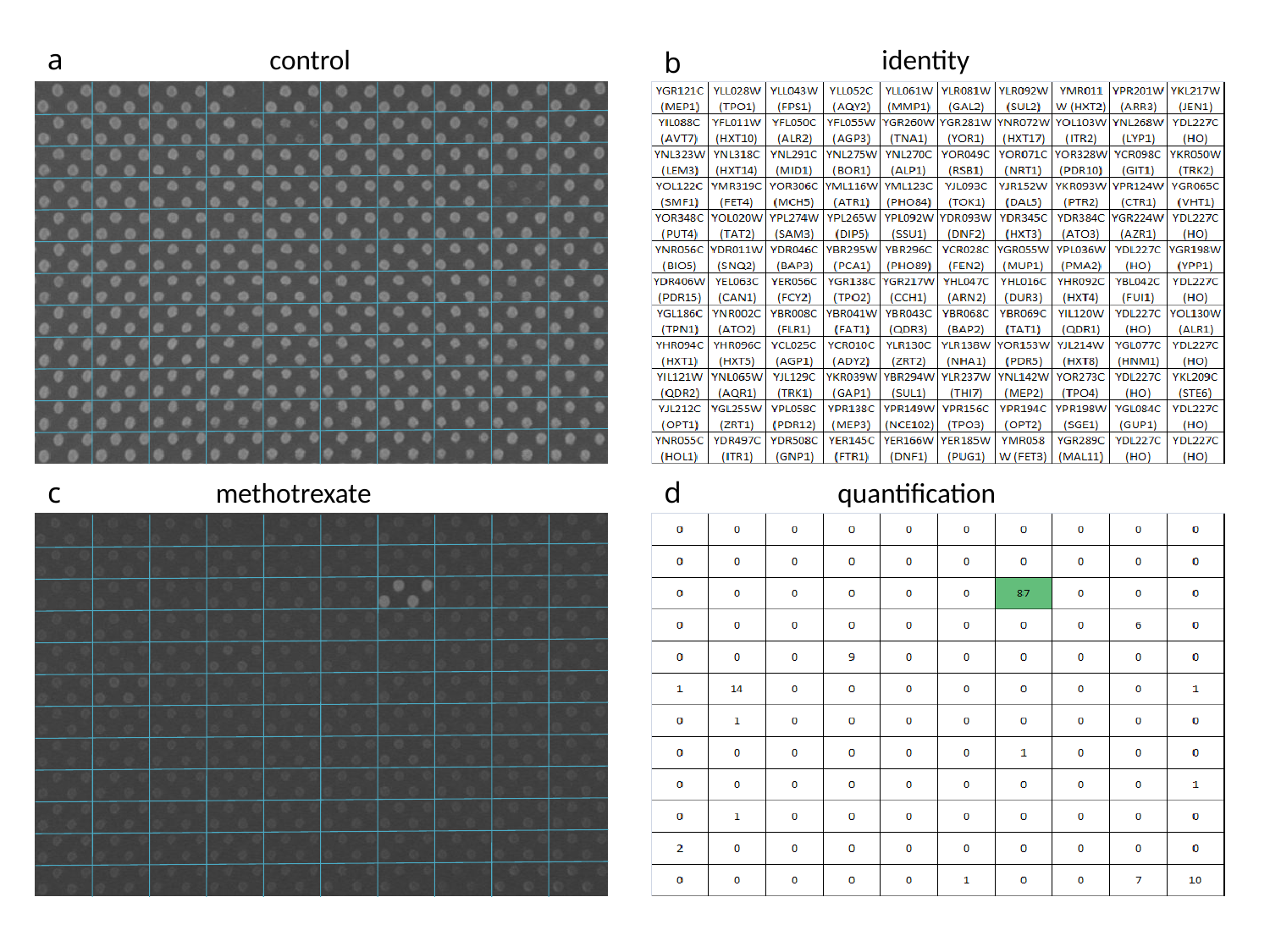

a
control
identity
b
c
methotrexate
d
quantification

Supplement: Additional file 4 — Identification of putative methotrexate transporters by the robot-assisted experiment. (a) Control plate with homozygous deletion mutant strains spotted in quadruplicates (by a Singer RoToR© HAD robot) onto F1 medium agar plate containing 1% DMSO. (b) Identity of deletion mutants spotted onto control and drug plates. (c) Homozygous deletion mutant strains spotted in quadruplicates (by a Singer RoToR© HAD robot) onto F1 medium agar plate containing 100 μM methotrexate. (d) Quantification of the relative growth between drug and control plates with methotrexate resistant strains highlighted in green. [file 1741-7007-9-70-S4.PPT]

## Slide 1
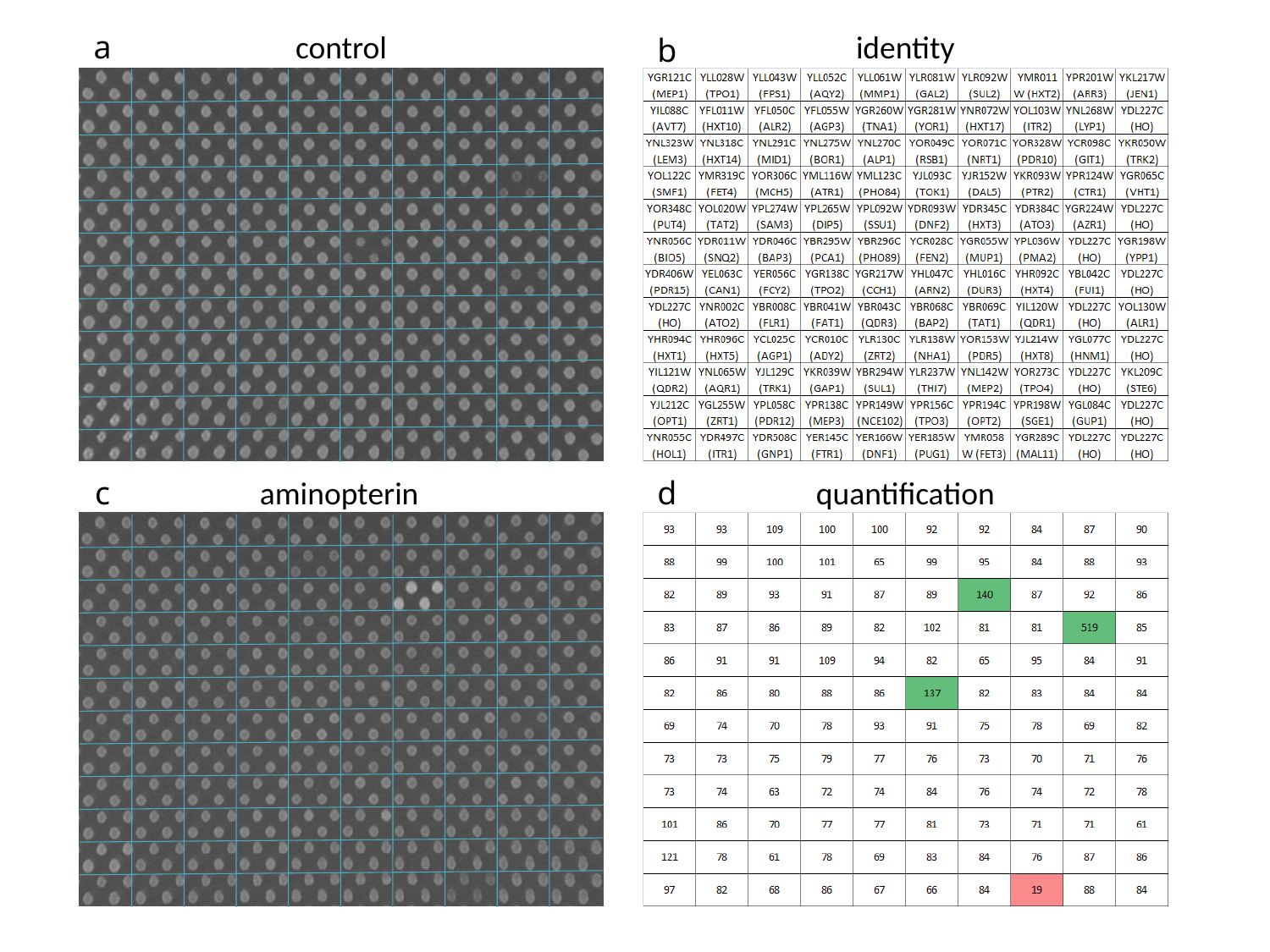

a
control
identity
b
c
aminopterin
d
quantification

Supplement: Additional file 5 — Identification of putative aminopterin transporters by the robot-assisted experiment. (a) Control plate with homozygous deletion mutant strains spotted in quadruplicates (by a Singer RoToR© HAD robot) onto F1 medium agar plate containing 1% DMSO. (b) Identity of deletion mutants spotted onto control and drug plates. (c) Homozygous deletion mutant strains spotted in quadruplicates (by a Singer RoToR© HAD robot) onto F1 medium agar plate containing 2 μM aminopterin. (d) Quantification of the relative growth between drug and control plates with aminopterin resistant strains highlighted in green. [file 1741-7007-9-70-S5.PPT]

## Slide 1
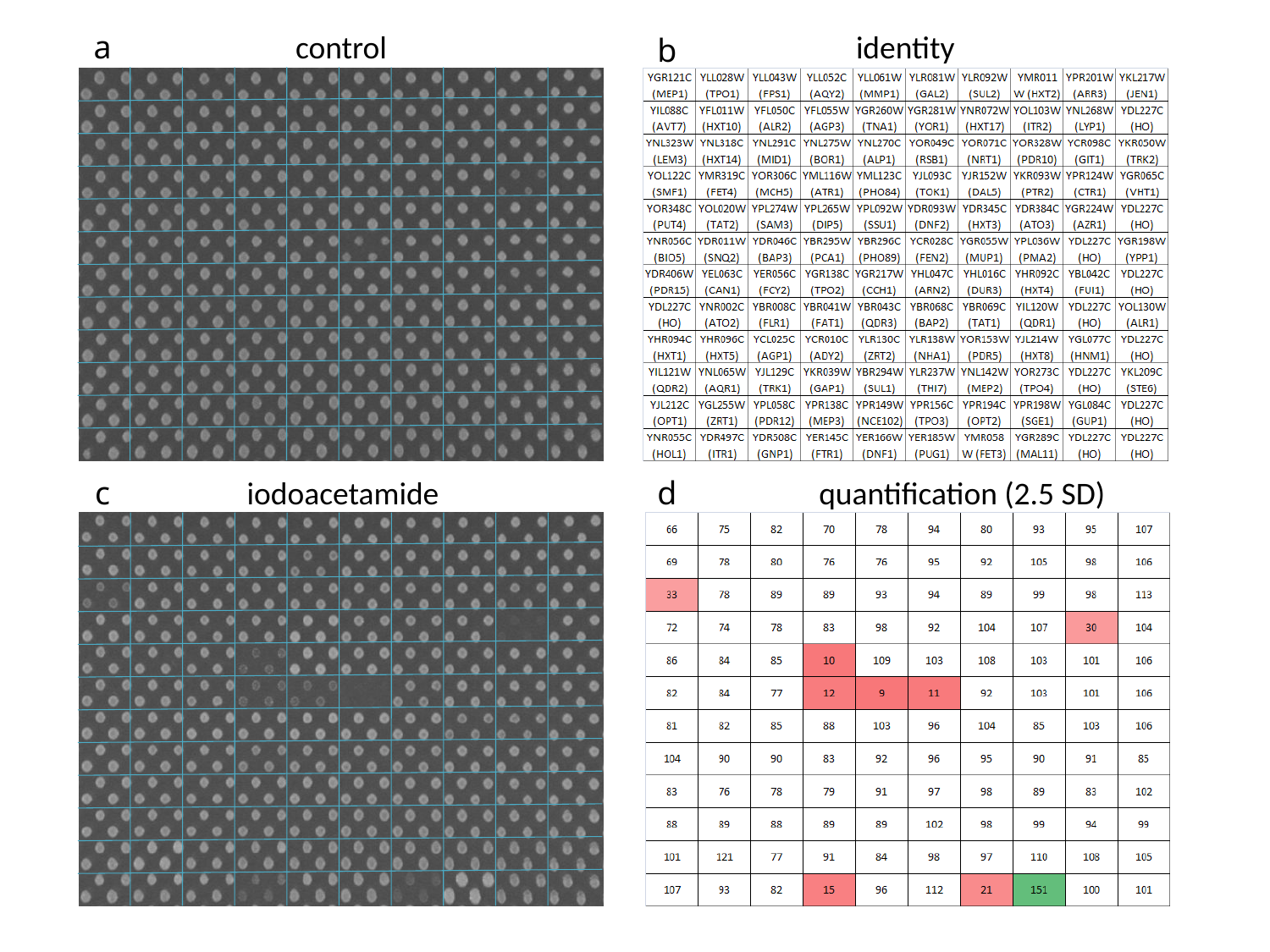

a
control
identity
b
c
iodoacetamide
d
quantification (2.5 SD)

Supplement: Additional file 6 — Identification of a putative iodoacetamide transporter by the robot-assisted experiment. (a) Control plate with homozygous deletion mutant strains spotted in quadruplicates (by a Singer RoToR© HAD robot) onto F1 medium agar plate containing 1% DMSO. (b) Identity of deletion mutants spotted onto control and drug plates. (c) Homozygous deletion mutant strains spotted in quadruplicates (by a Singer RoToR© HAD robot) onto F1 medium agar plate containing 20 μM iodoacetamide. (d) Quantification of the relative growth between drug and control plates with iodoacetamide resistant strains highlighted in green. [file 1741-7007-9-70-S6.PPT]

## Slide 1
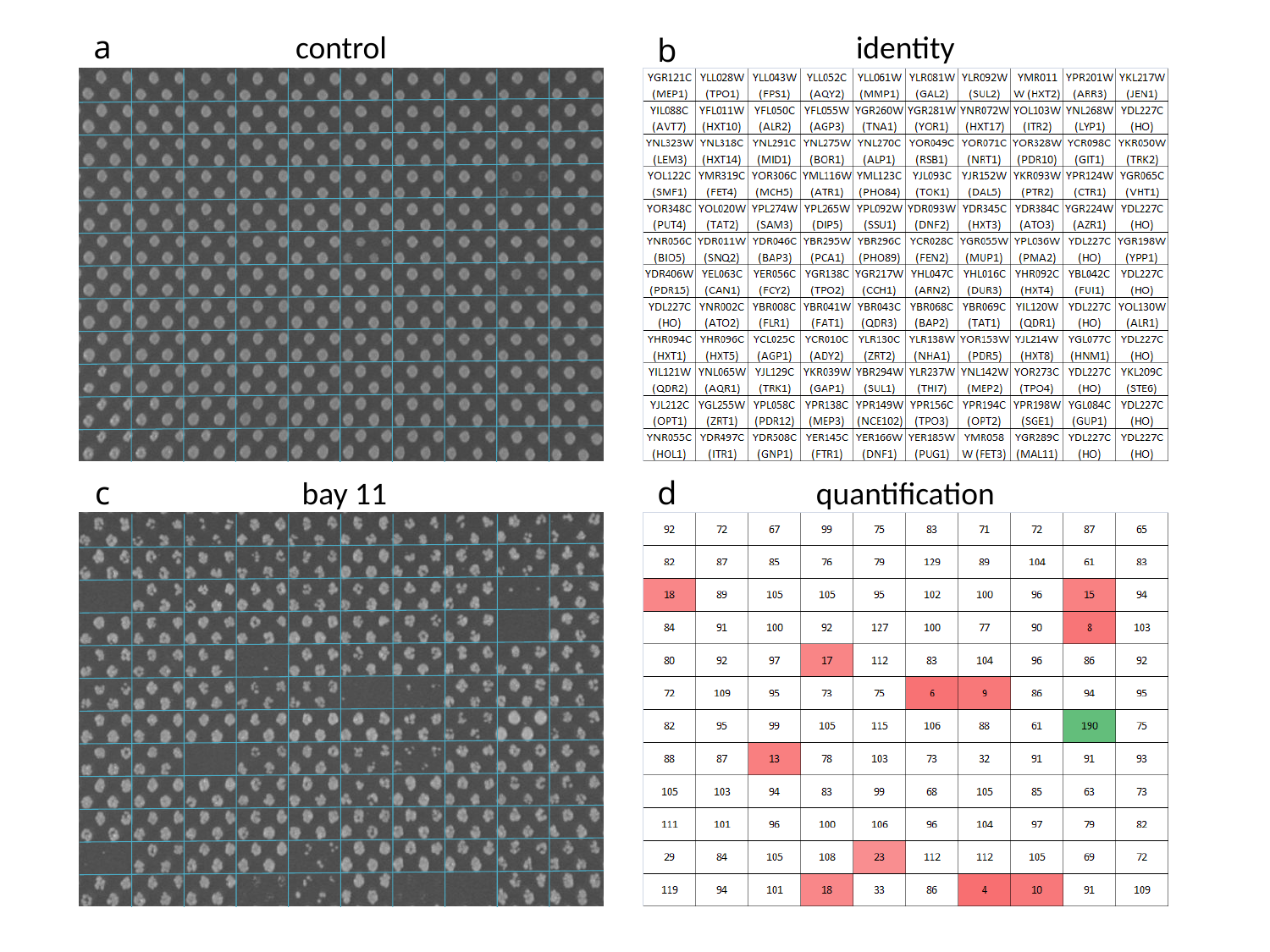

a
control
identity
b
c
bay 11
d
quantification

Supplement: Additional file 7 — Identification of putative Bay11-7085 transporters by the robot-assisted experiment. (a) Control plate with homozygous deletion mutant strains spotted in quadruplicates (by a Singer RoToR© HAD robot) onto F1 medium agar plate containing 1% DMSO. (b) Identity of deletion mutants spotted onto control and drug plates. (c) Homozygous deletion mutant strains spotted in quadruplicates (by a Singer RoToR© HAD robot) onto F1 medium agar plate containing 10 μM Bay11-7085. (d) Quantification of the relative growth between drug and control plates with Bay11-7085 resistant strains highlighted in green. [file 1741-7007-9-70-S7.PPT]

## Slide 1
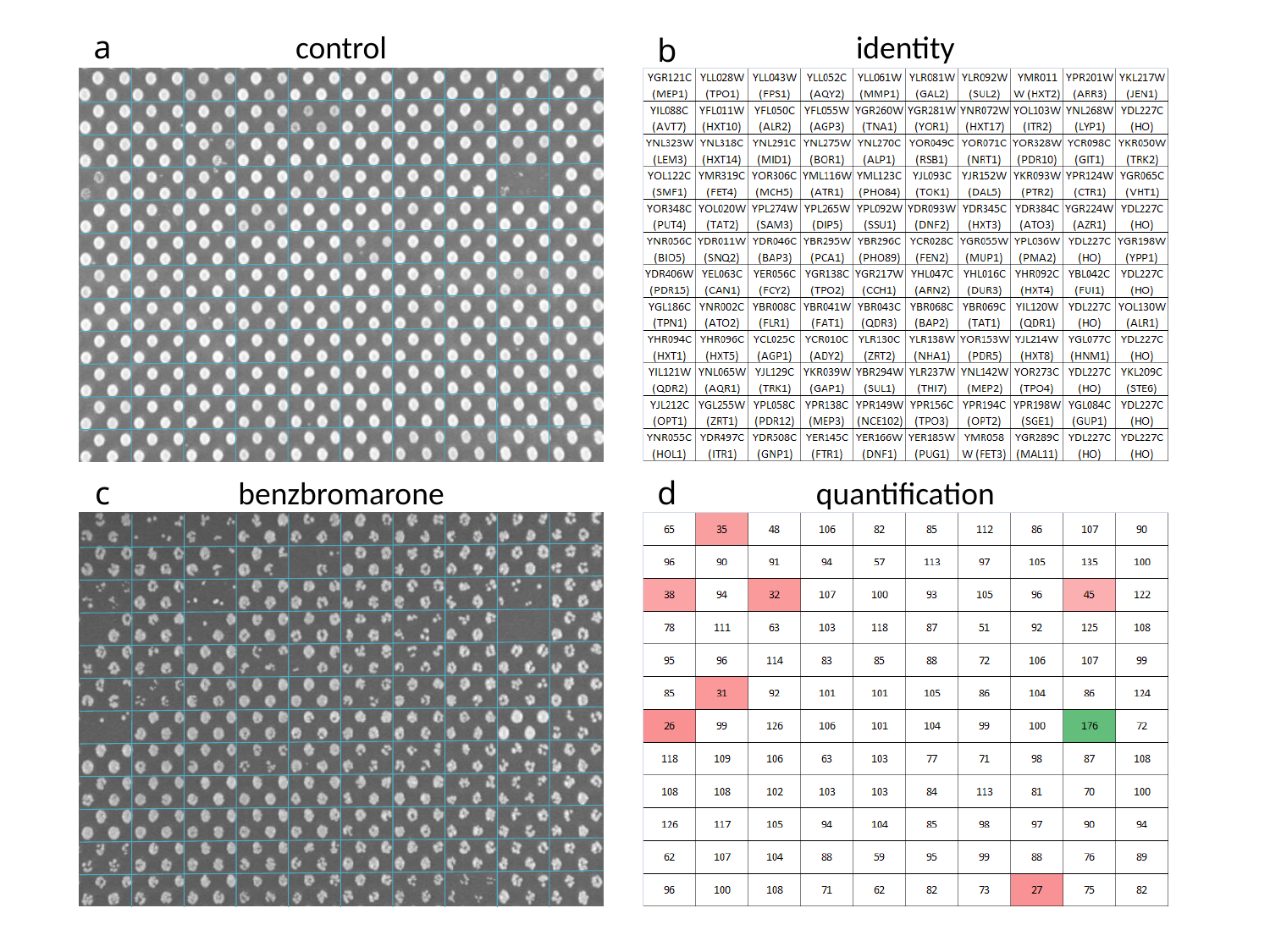

a
control
identity
b
c
benzbromarone
d
quantification

Supplement: Additional file 8 — Identification of putative benzbromarone transporters by the robot-assisted experiment. (a) Control plate with homozygous deletion mutant strains spotted in quadruplicates (by a Singer RoToR© HAD robot) onto F1 medium agar plate containing 1% DMSO. (b) Identity of deletion mutants spotted onto control and drug plates. (c) Homozygous deletion mutant strains spotted in quadruplicates (by a Singer RoToR© HAD robot) onto F1 medium agar plate containing 2 μM benzbromarone. (d) Quantification of the relative growth between drug and control plates with benzbromarone resistant strains highlighted in green. [file 1741-7007-9-70-S8.PPT]

## Slide 1
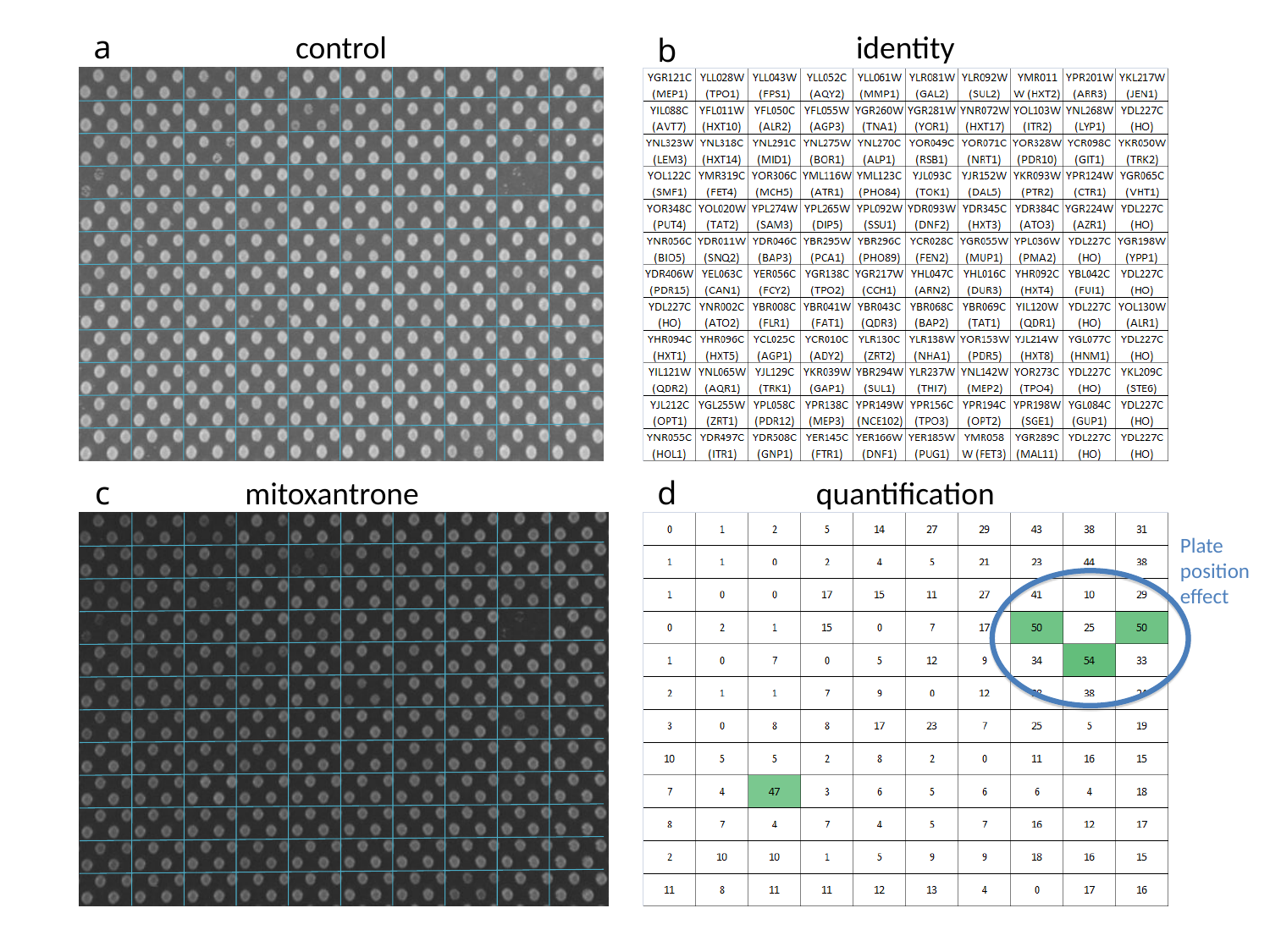

a
control
identity
b
c
mitoxantrone
d
quantification
Plate
position effect

Supplement: Additional file 9 — Identification of putative mitoxantrone transporters by the robot-assisted experiment. (a) Control plate with homozygous deletion mutant strains spotted in quadruplicates (by a Singer RoToR© HAD robot) onto F1 medium agar plate containing 1% DMSO. (b) Identity of deletion mutants spotted onto control and drug plates. (c) Homozygous deletion mutant strains spotted in quadruplicates (by a Singer RoToR© HAD robot) onto F1 medium agar plate containing 75 μM mitoxantrone. (d) Quantification of the relative growth between drug and control plates with mitoxantrone resistant strains highlighted in green. [file 1741-7007-9-70-S9.PPT]

## Slide 1
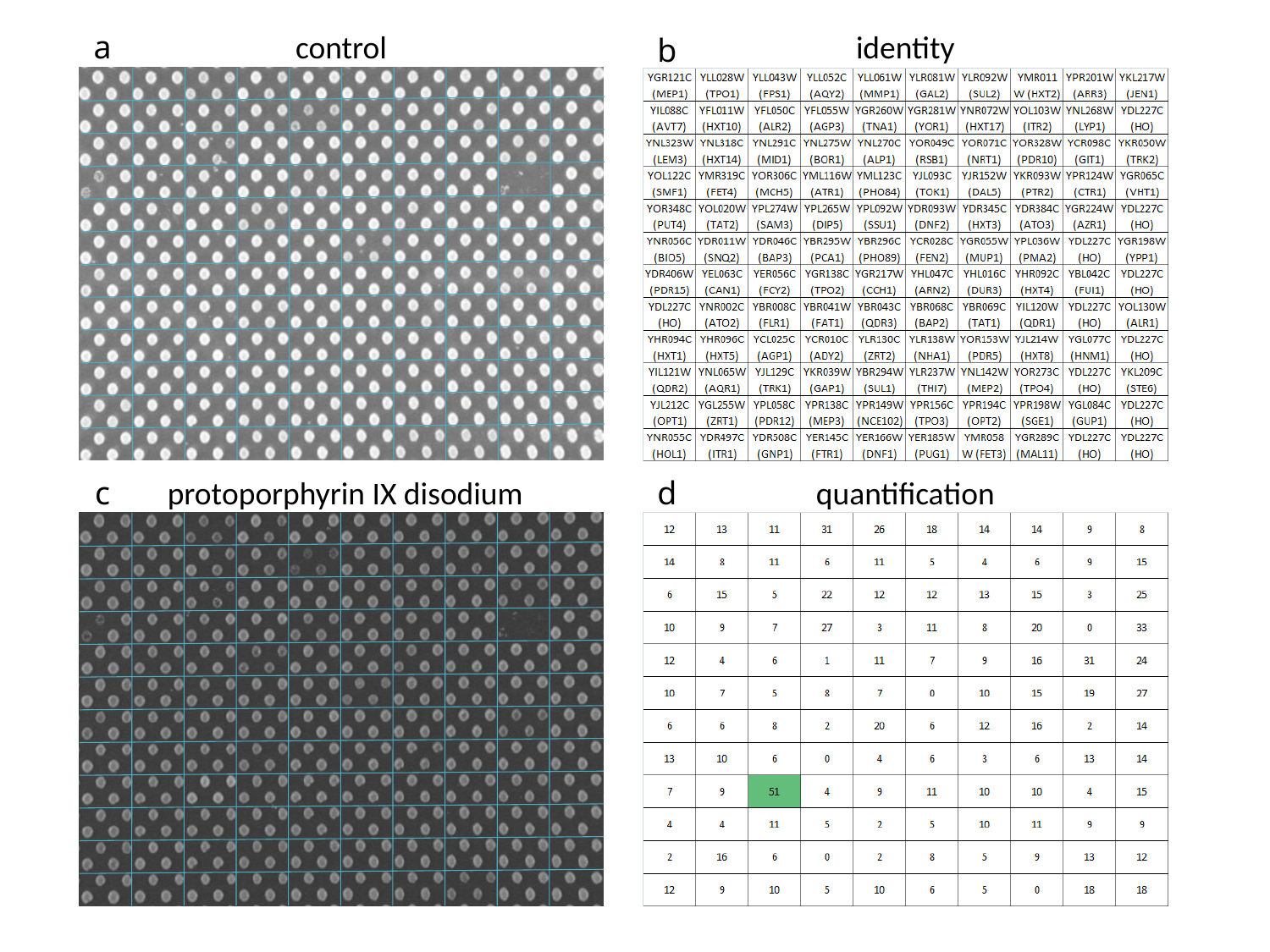

a
control
identity
b
c
protoporphyrin IX disodium
d
quantification

Supplement: Additional file 10 — Identification of putative protoporphyrin transporters by the robot-assisted experiment. (a) Control plate with homozygous deletion mutant strains spotted in quadruplicates (by a Singer RoToR© HAD robot) onto F1 medium agar plate containing 1% DMSO. (b) Identity of deletion mutants spotted onto control and drug plates. (c) Homozygous deletion mutant strains spotted in quadruplicates (by a Singer RoToR© HAD robot) onto F1 medium agar plate containing 600 μM protoporphyrin. (d) Quantification of the relative growth between drug and control plates with protoporphyrin resistant strains highlighted in green. [file 1741-7007-9-70-S10.PPT]

## Slide 1
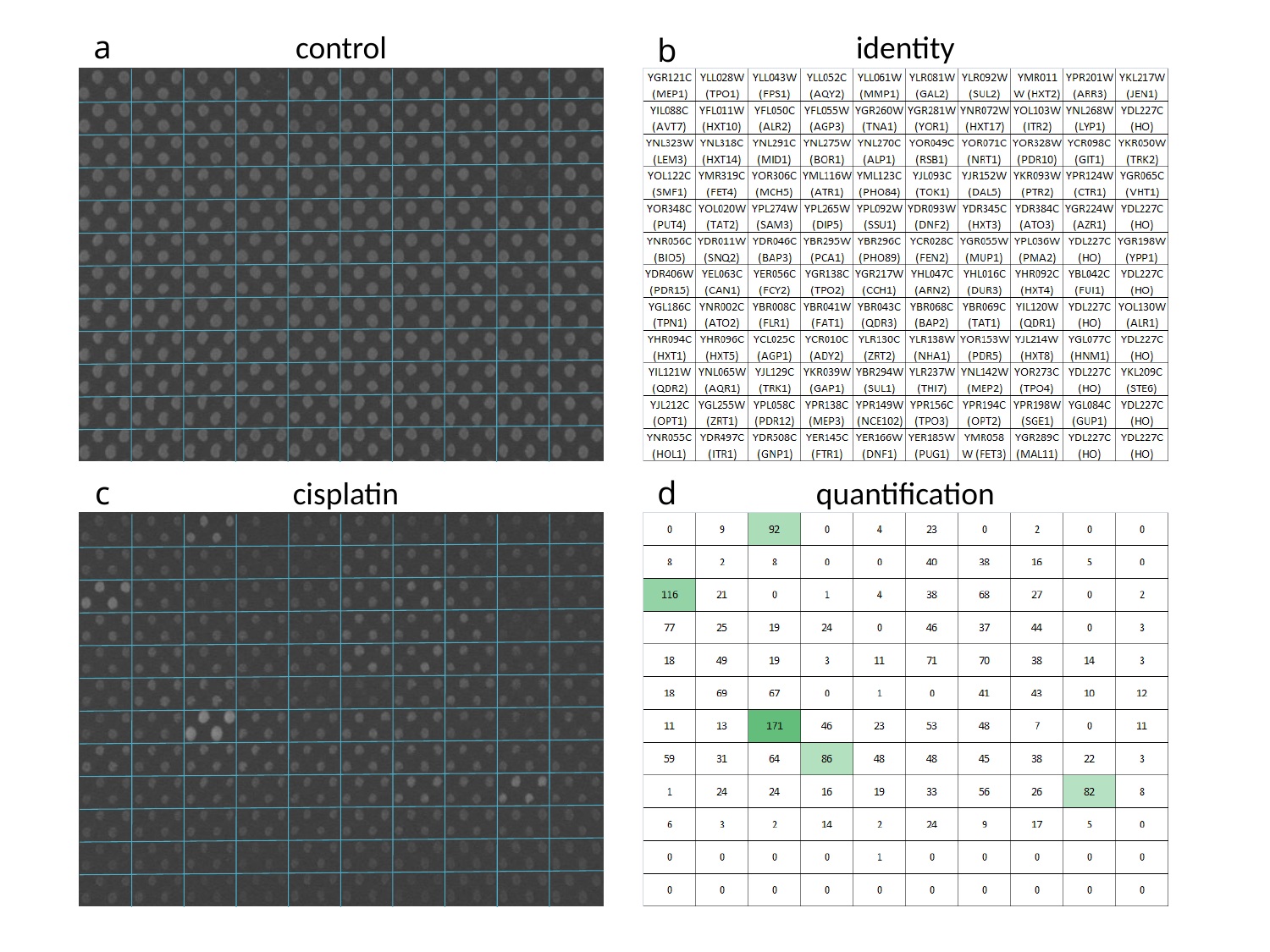

a
control
identity
b
c
cisplatin
d
quantification

Supplement: Additional file 11 — Identification of putative cisplatin transporters by the robot-assisted experiment. (a) Control plate with homozygous deletion mutant strains spotted in quadruplicates (by a Singer RoToR© HAD robot) onto F1 medium agar plate. (b) Identity of deletion mutants spotted onto control and drug plates. (c) Homozygous deletion mutant strains spotted in quadruplicates (by a Singer RoToR© HAD robot) onto F1 medium agar plate containing 50 μM cisplatin. (d) Quantification of the relative growth between drug and control plates with cisplatin resistant strains highlighted in green (3 SD above the plate average). [file 1741-7007-9-70-S11.PPT]

## Slide 1
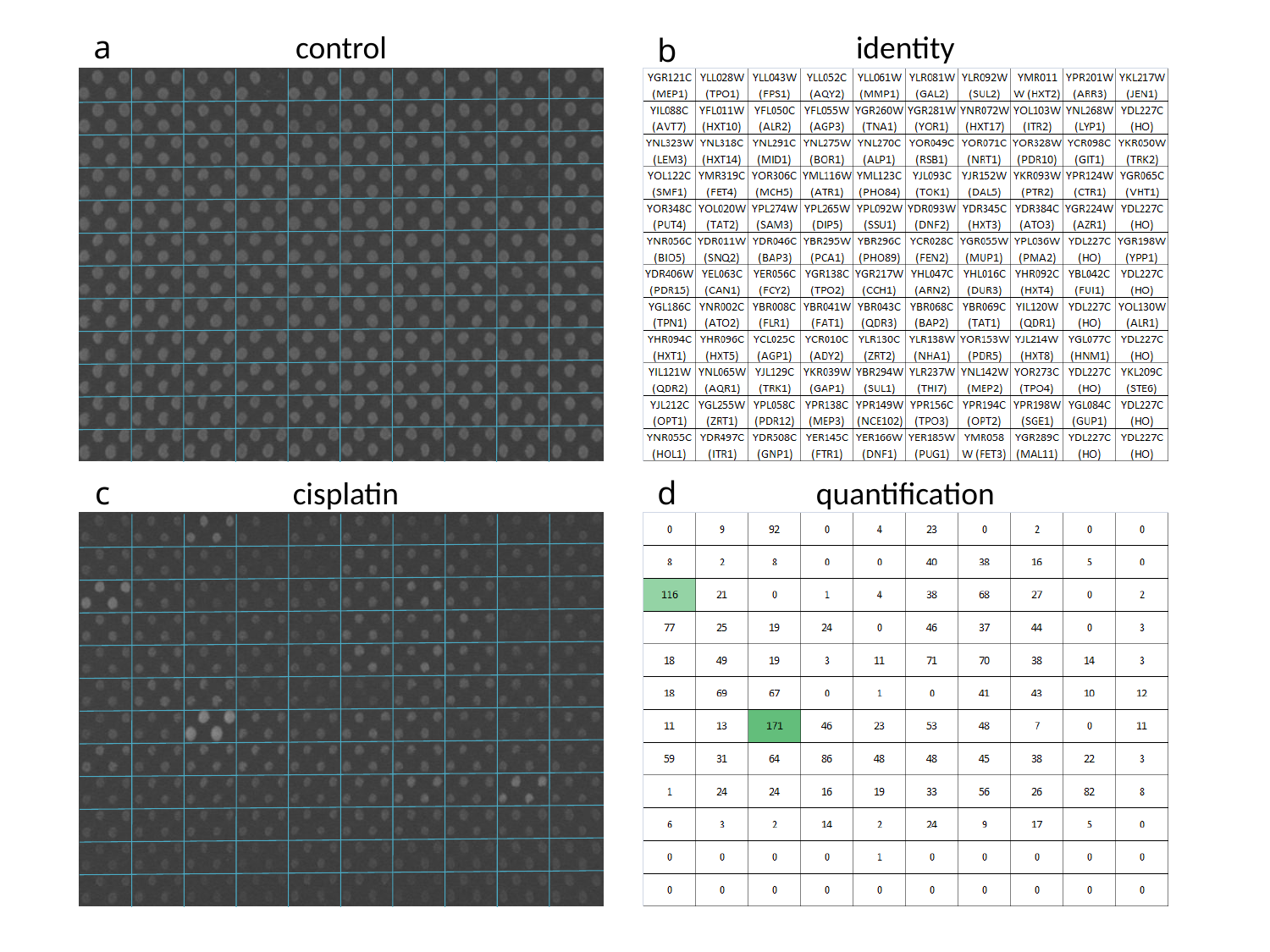

a
control
identity
b
c
cisplatin
d
quantification

Supplement: Additional file 12 — Identification of putative cisplatin transporters by the robot-assisted experiment. (a) Control plate with homozygous deletion mutant strains spotted in quadruplicates (by a Singer RoToR© HAD robot) onto F1 medium agar plate containing 1% DMSO. (b) Identity of deletion mutants spotted onto control and drug plates. (c) Homozygous deletion mutant strains spotted in quadruplicates (by a Singer RoToR© HAD robot) onto F1 medium agar plate containing 50 μM cisplatin. (d) Quantification of the relative growth between drug and control plates with cisplatin resistant strains highlighted in green (2 SD above the plate average). [file 1741-7007-9-70-S12.PPT]

## Slide 1
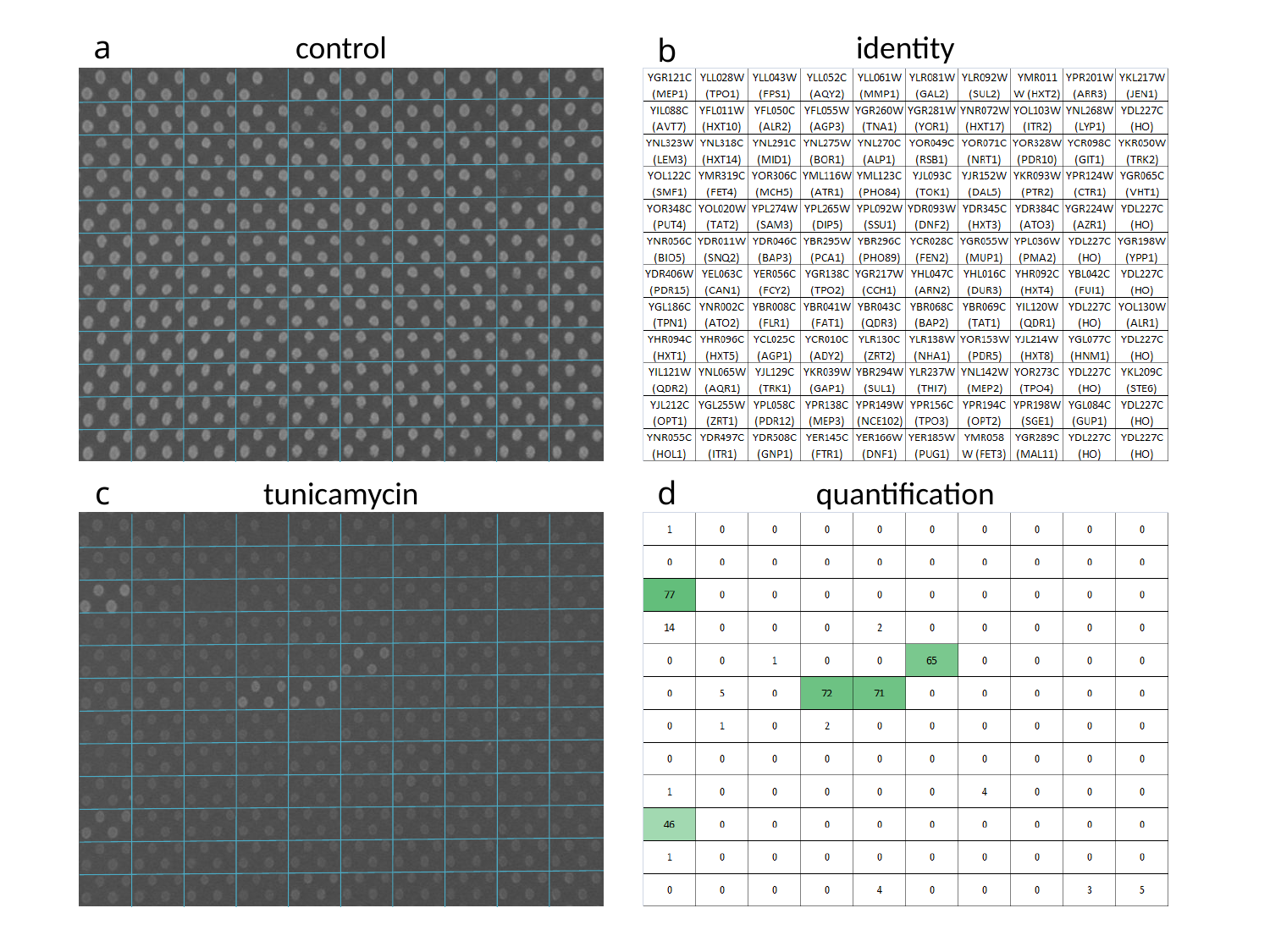

a
control
identity
b
c
tunicamycin
d
quantification

Supplement: Additional file 13 — Identification of putative tunicamycin transporters by the robot-assisted experiment. (a) Control plate with homozygous deletion mutant strains spotted in quadruplicates (by a Singer RoToR© HAD robot) onto F1 medium agar plate containing 1% DMSO. (b) Identity of deletion mutants spotted onto control and drug plates. (c) Homozygous deletion mutant strains spotted in quadruplicates (by a Singer RoToR© HAD robot) onto F1 medium agar plate containing 4 μM tunicamycin. (d) Quantification of the relative growth between drug and control plates with tunicamycin resistant strains highlighted in green. [file 1741-7007-9-70-S13.PPT]

## Slide 1
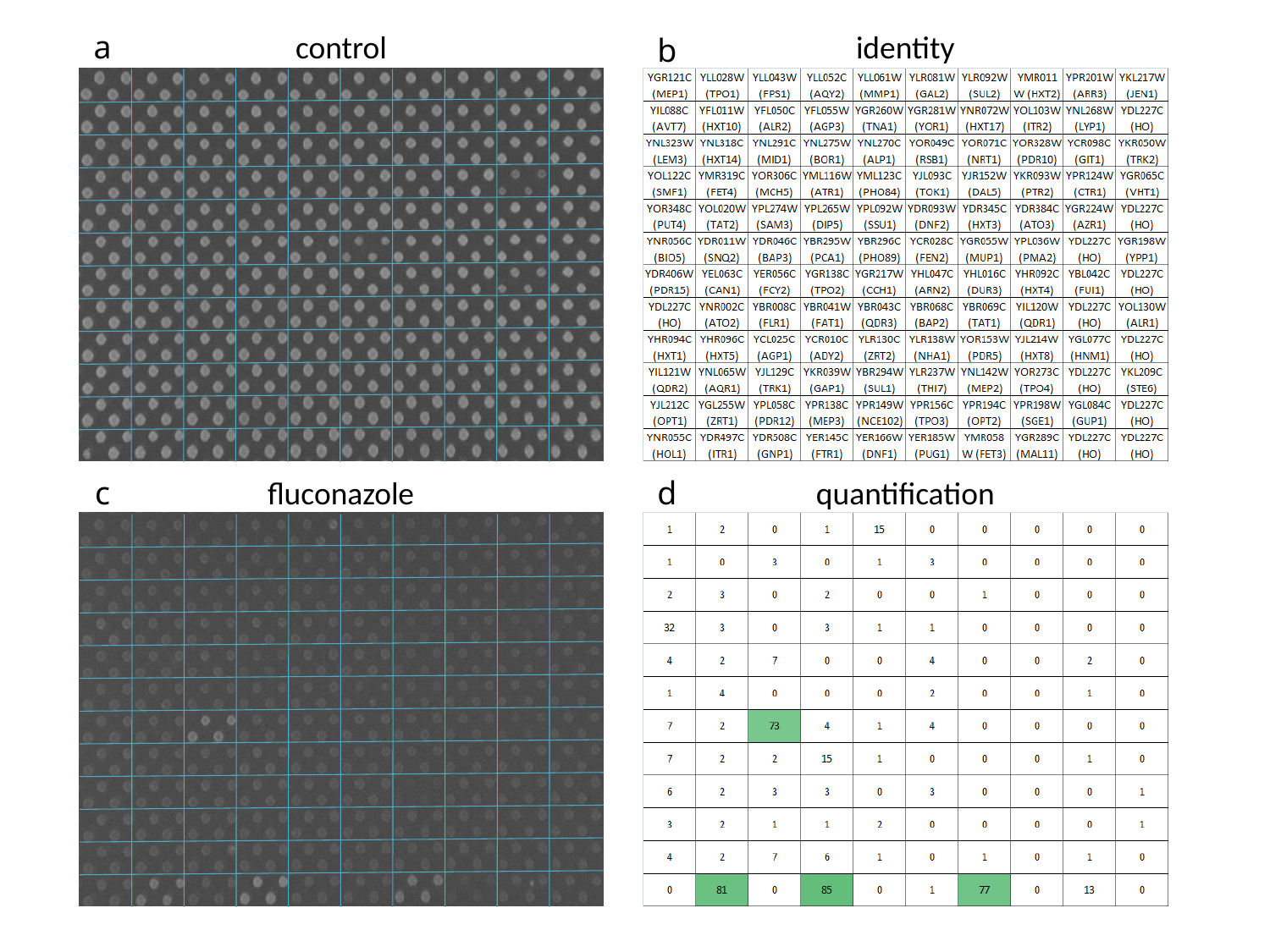

a
control
identity
b
c
fluconazole
d
quantification

Supplement: Additional file 15 — Identification of putative fluconazole transporters by the robot-assisted experiment. (a) Control plate with homozygous deletion mutant strains spotted in quadruplicates (by a Singer RoToR© HAD robot) onto F1 medium agar plate containing 1% DMSO. (b) Identity of deletion mutants spotted onto control and drug plates. (c) Homozygous deletion mutant strains spotted in quadruplicates (by a Singer RoToR© HAD robot) onto F1 medium agar plate containing 100 μM fluconazole. (d) Quantification of the relative growth between drug and control plates with fluconazole resistant strains highlighted in green. [file 1741-7007-9-70-S15.PPT]

## Slide 1
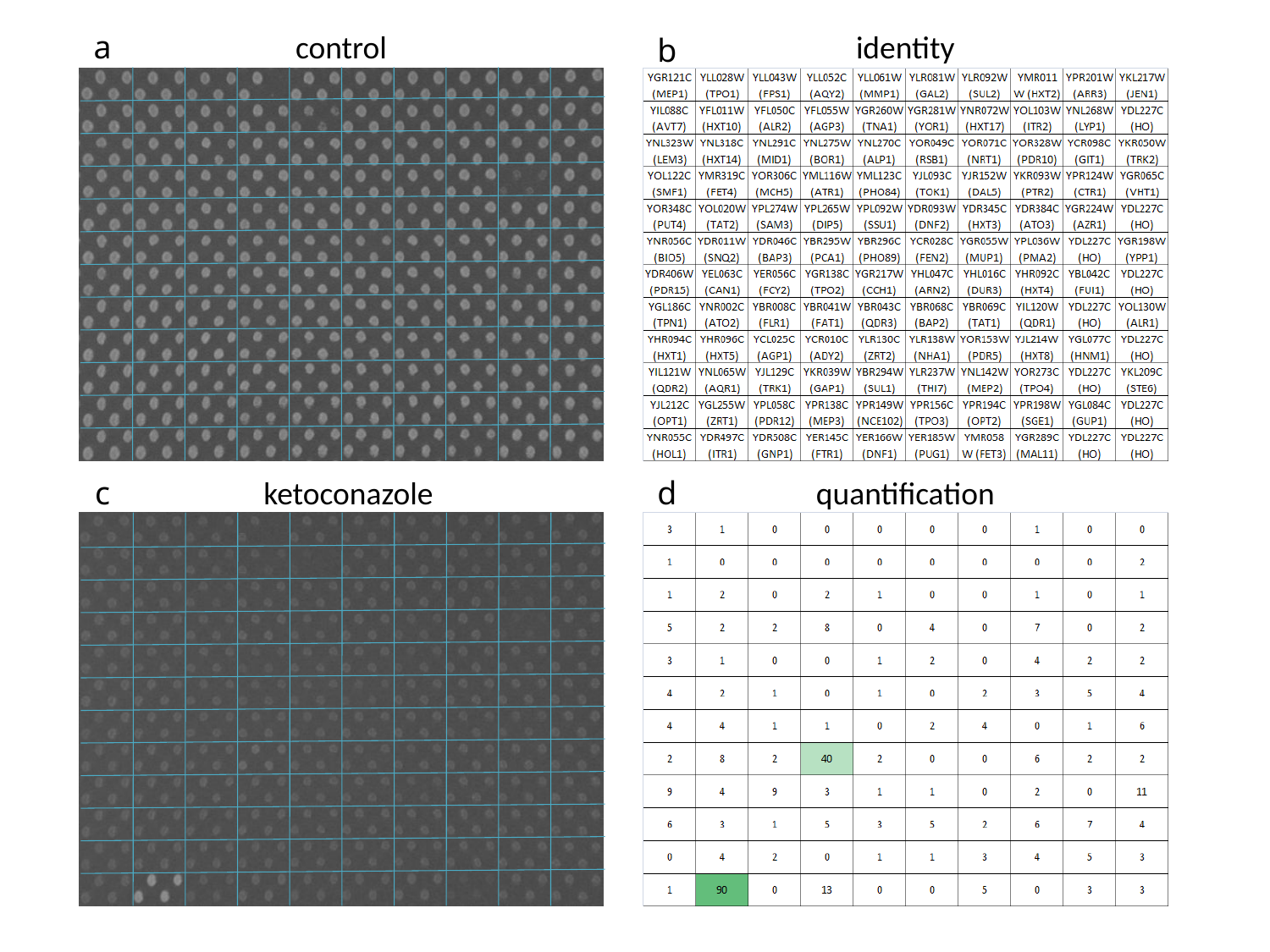

a
control
identity
b
c
ketoconazole
d
quantification

Supplement: Additional file 16 — Identification of putative ketoconazole transporters by the robot-assisted experiment. (a) Control plate with homozygous deletion mutant strains spotted in quadruplicates (by a Singer RoToR© HAD robot) onto F1 medium agar plate containing 1% DMSO. (b) Identity of deletion mutants spotted onto control and drug plates. (c) Homozygous deletion mutant strains spotted in quadruplicates (by a Singer RoToR© HAD robot) onto F1 medium agar plate containing 80 μM ketoconazole. (d) Quantification of the relative growth between drug and control plates with ketoconazole resistant strains highlighted in green. [file 1741-7007-9-70-S16.PPT]

## Slide 1
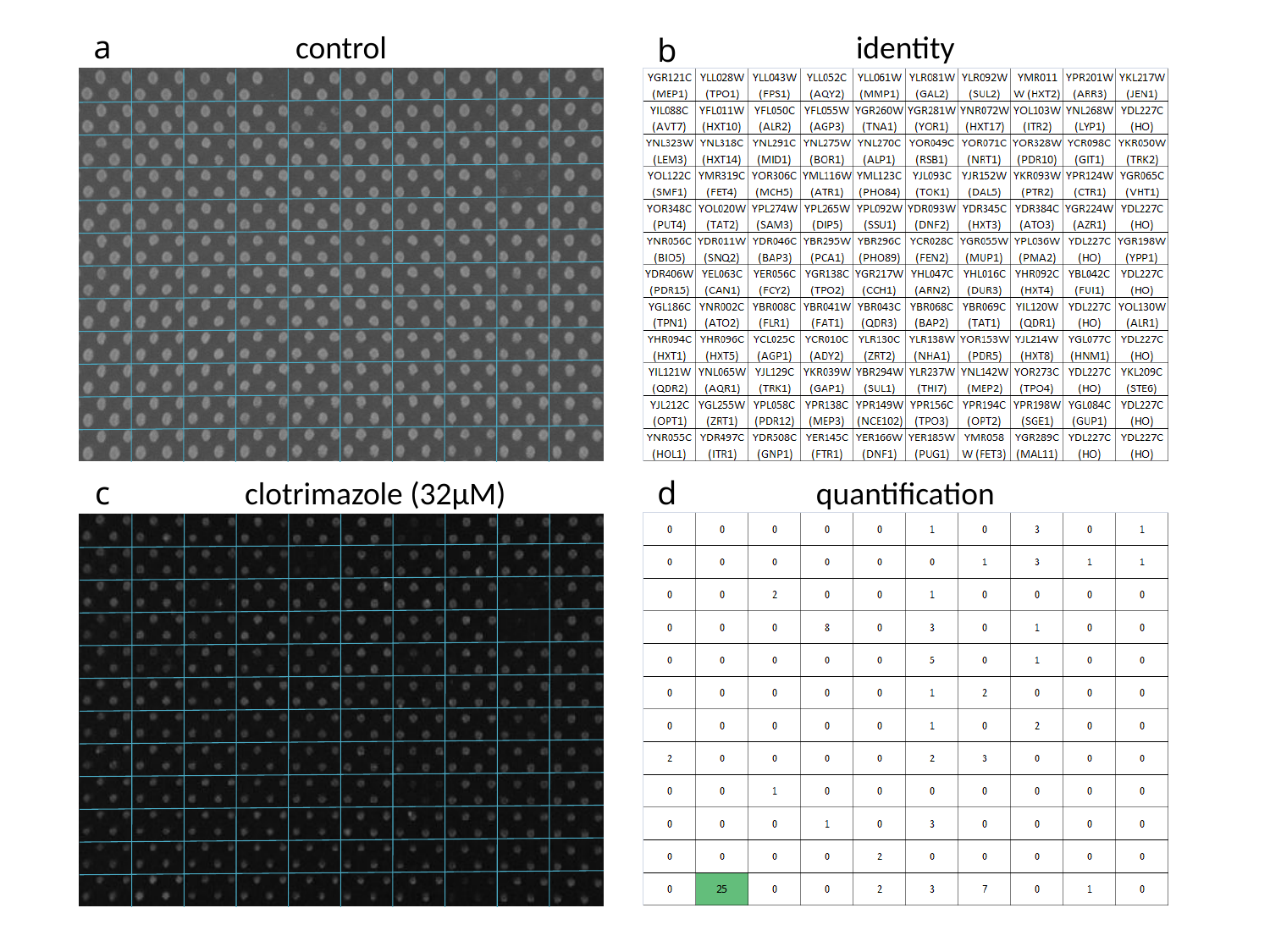

a
control
identity
b
c
clotrimazole (32μM)
d
quantification

Supplement: Additional file 17 — Identification of putative clotrimazole transporters by the robot-assisted experiment. (a) Control plate with homozygous deletion mutant strains spotted in quadruplicates (by a Singer RoToR© HAD robot) onto F1 medium agar plate containing 1% DMSO. (b) Identity of deletion mutants spotted onto control and drug plates. (c) Homozygous deletion mutant strains spotted in quadruplicates (by a Singer RoToR© HAD robot) onto F1 medium agar plate containing 32 μM clotrimazole. (d) Quantification of the relative growth between drug and control plates with clotrimazole resistant strains highlighted in green. [file 1741-7007-9-70-S17.PPT]

## Slide 1
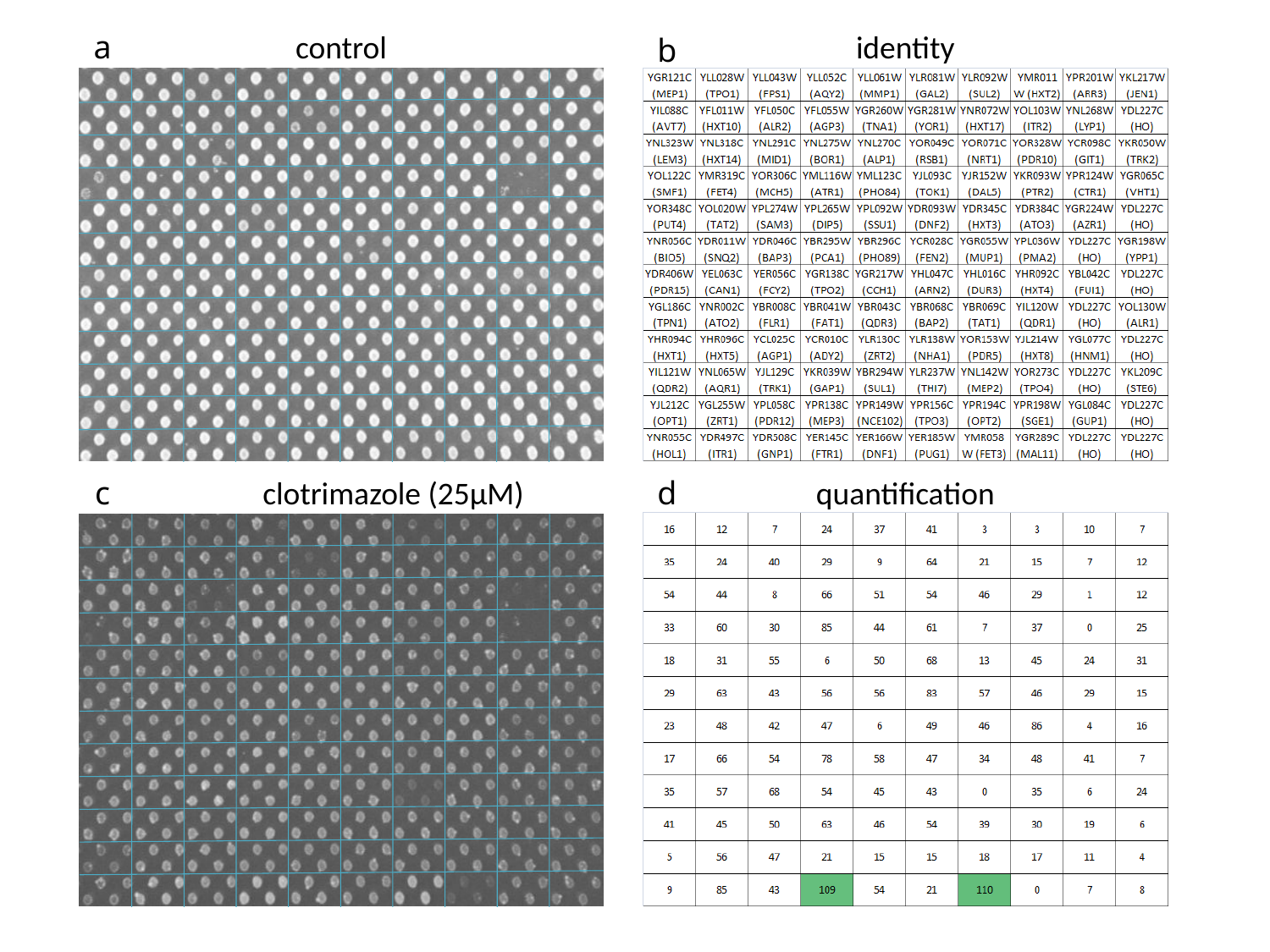

a
control
identity
b
c
clotrimazole (25μM)
d
quantification

Supplement: Additional file 18 — Identification of putative clotrimazole transporters by the robot-assisted experiment. (a) Control plate with homozygous deletion mutant strains spotted in quadruplicates (by a Singer RoToR© HAD robot) onto F1 medium agar plate containing 1% DMSO. (b) Identity of deletion mutants spotted onto control and drug plates. (c) Homozygous deletion mutant strains spotted in quadruplicates (by a Singer RoToR© HAD robot) onto F1 medium agar plate containing 25 μM clotrimazole. (d) Quantification of the relative growth between drug and control plates with clotrimazole resistant strains highlighted in green. [file 1741-7007-9-70-S18.PPT]

## Slide 1
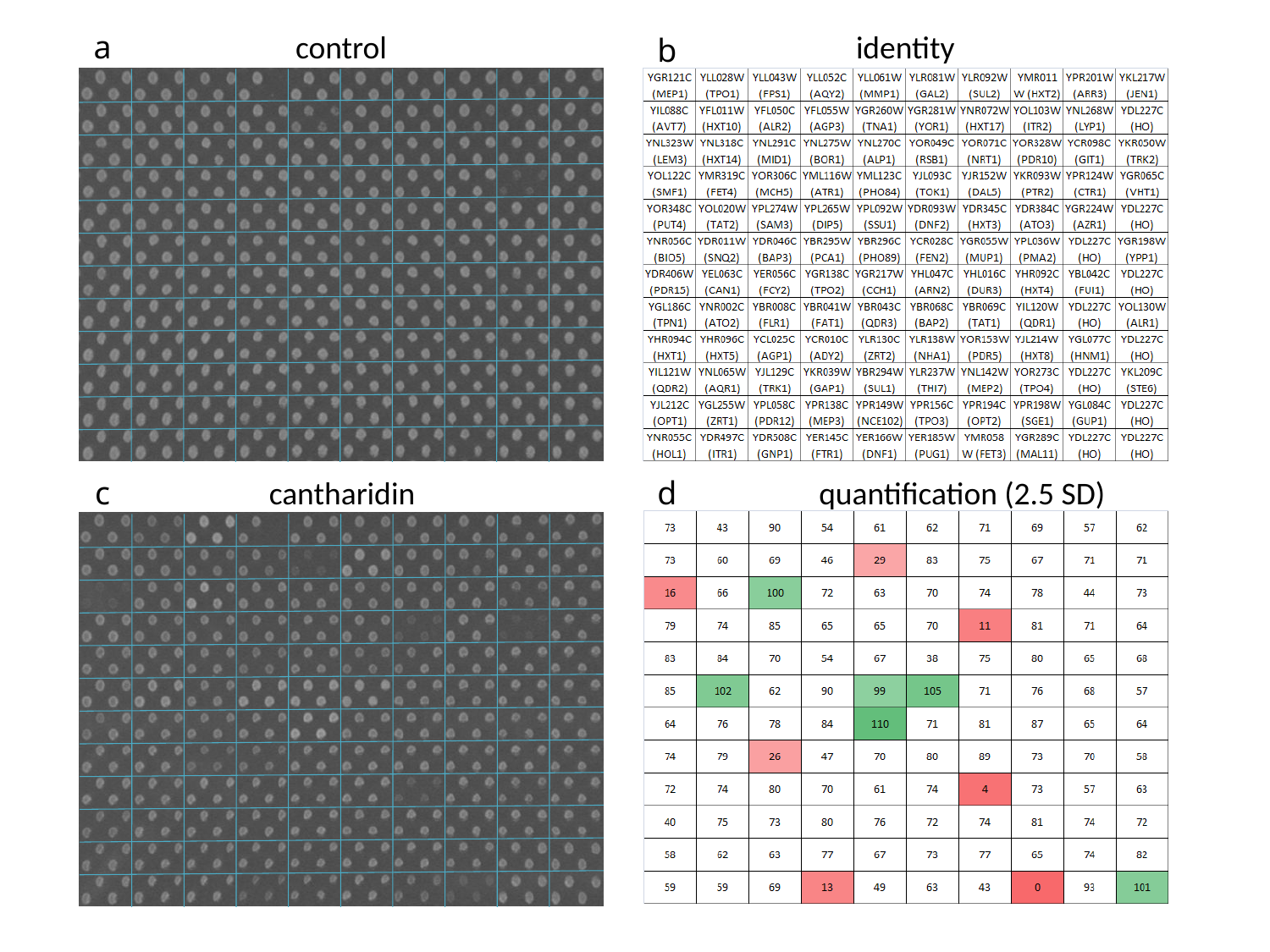

a
control
identity
b
c
cantharidin
d
quantification (2.5 SD)

Supplement: Additional file 19 — Identification of putative cantharidin transporters by the robot-assisted experiment. (a) Control plate with homozygous deletion mutant strains spotted in quadruplicates (by a Singer RoToR© HAD robot) onto F1 medium agar plate containing 1% DMSO. (b) Identity of deletion mutants spotted onto control and drug plates. (c) Homozygous deletion mutant strains spotted in quadruplicates (by a Singer RoToR© HAD robot) onto F1 medium agar plate containing 30 μM cantharidin. (d) Quantification of the relative growth between drug and control plates with cantharidin resistant strains highlighted in green (2.5 standard deviations above the plate average). [file 1741-7007-9-70-S19.PPT]

## Slide 1
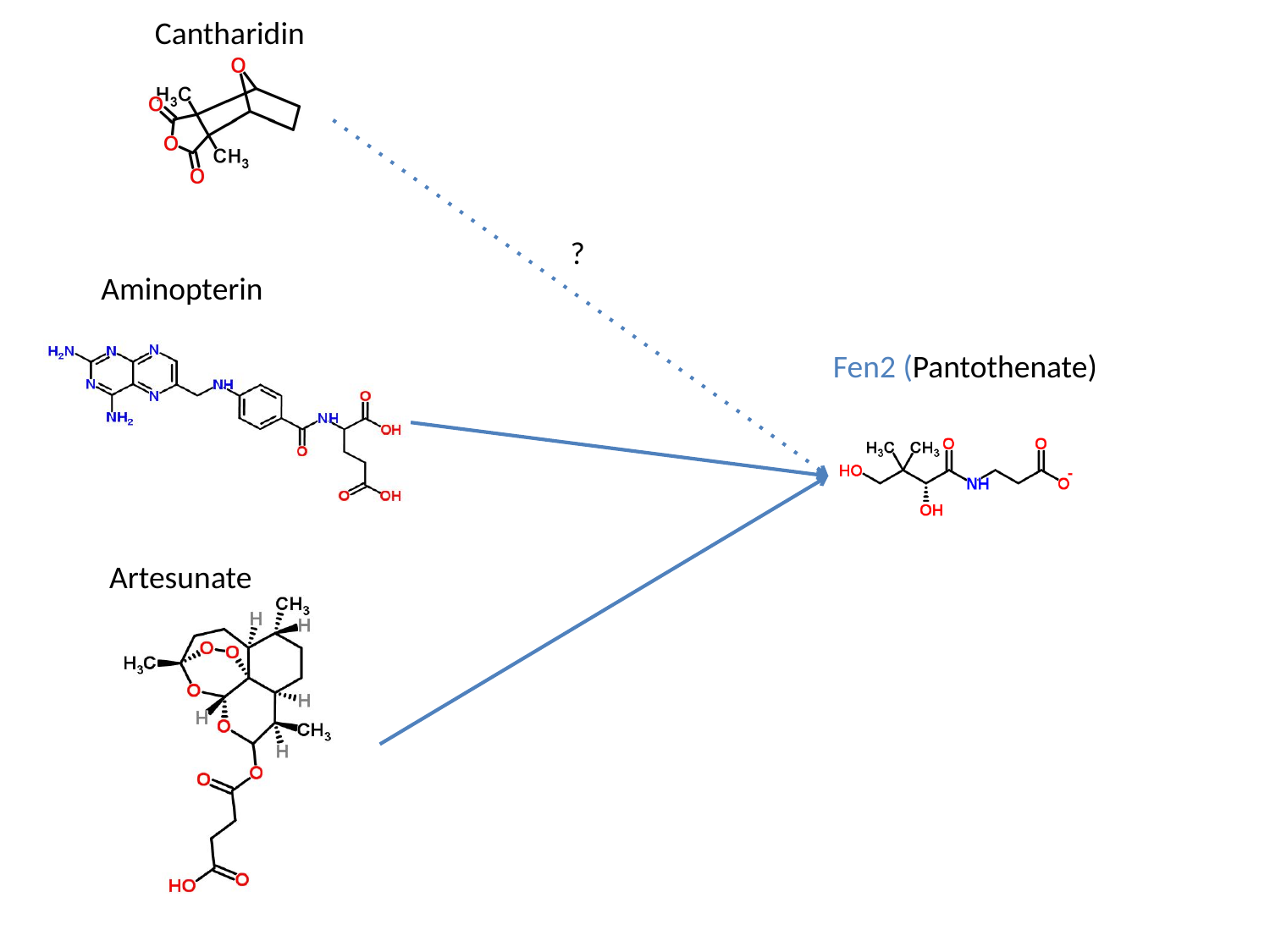

Cantharidin
?
Aminopterin
Fen2 (Pantothenate)
Artesunate

Supplement: Additional file 20 — Chemical structures of cantharidin, artesunate and aminopterin as well as that of the native substrate carried by the proposed transporter, Fen2p. [file 1741-7007-9-70-S20.PPT]

## Slide 1
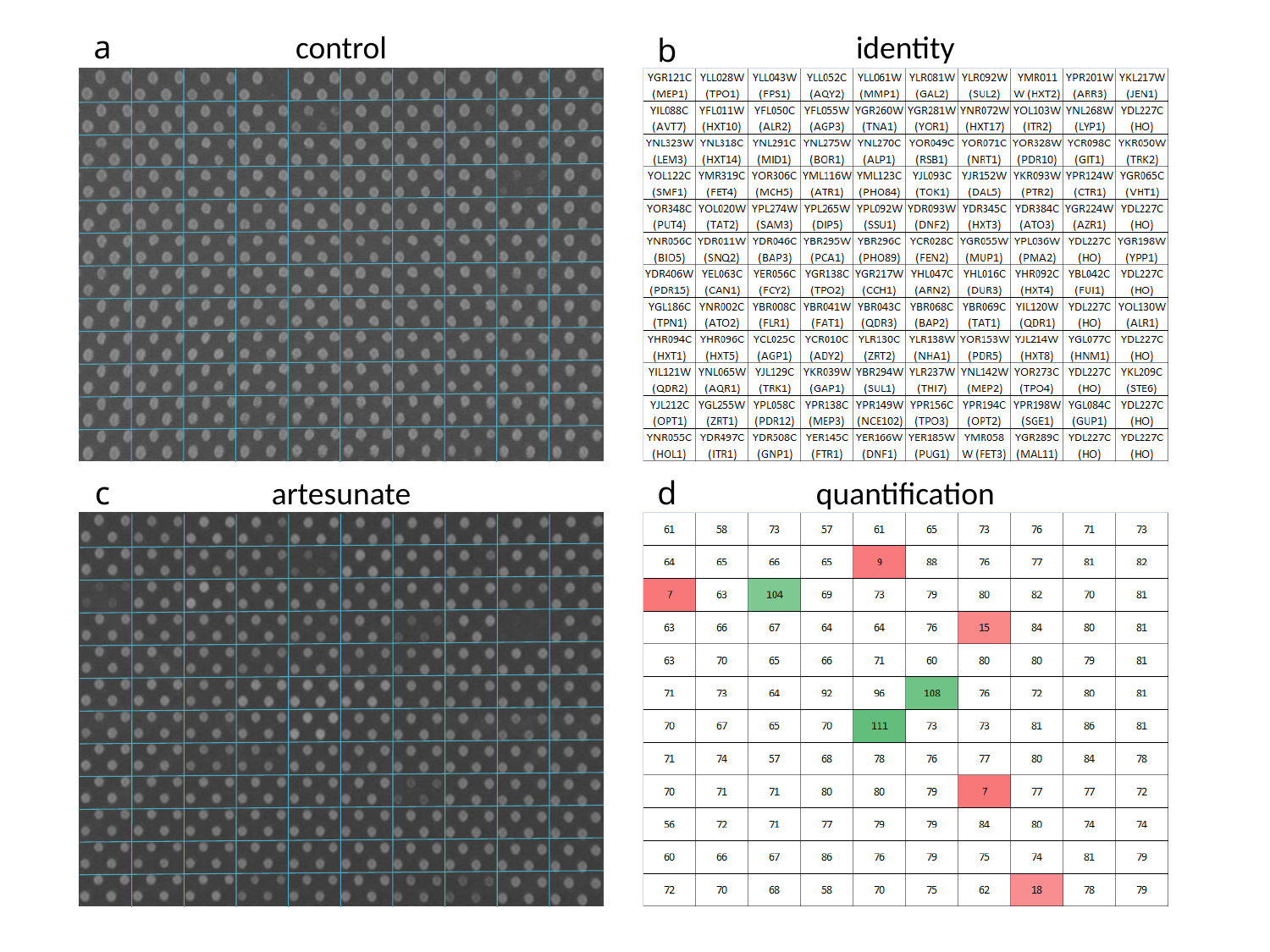

a
control
identity
b
c
artesunate
d
quantification

Supplement: Additional file 21 — Identification of putative artesunate transporters by the robot-assisted experiment. (a) Control plate with homozygous deletion mutant strains spotted in quadruplicates (by a Singer RoToR© HAD robot) onto F1 medium agar plate containing 1% DMSO. (b. Identity of deletion mutants spotted onto control and drug plates. (c) Homozygous deletion mutant strains spotted in quadruplicates (by a Singer RoToR© HAD robot) onto F1 medium agar plate containing 100 μM artesunate. (d) Quantification of the relative growth between drug and control plates with artesunate resistant strains highlighted in green (2 SD above the plate average). [file 1741-7007-9-70-S21.PPT]

## Slide 1
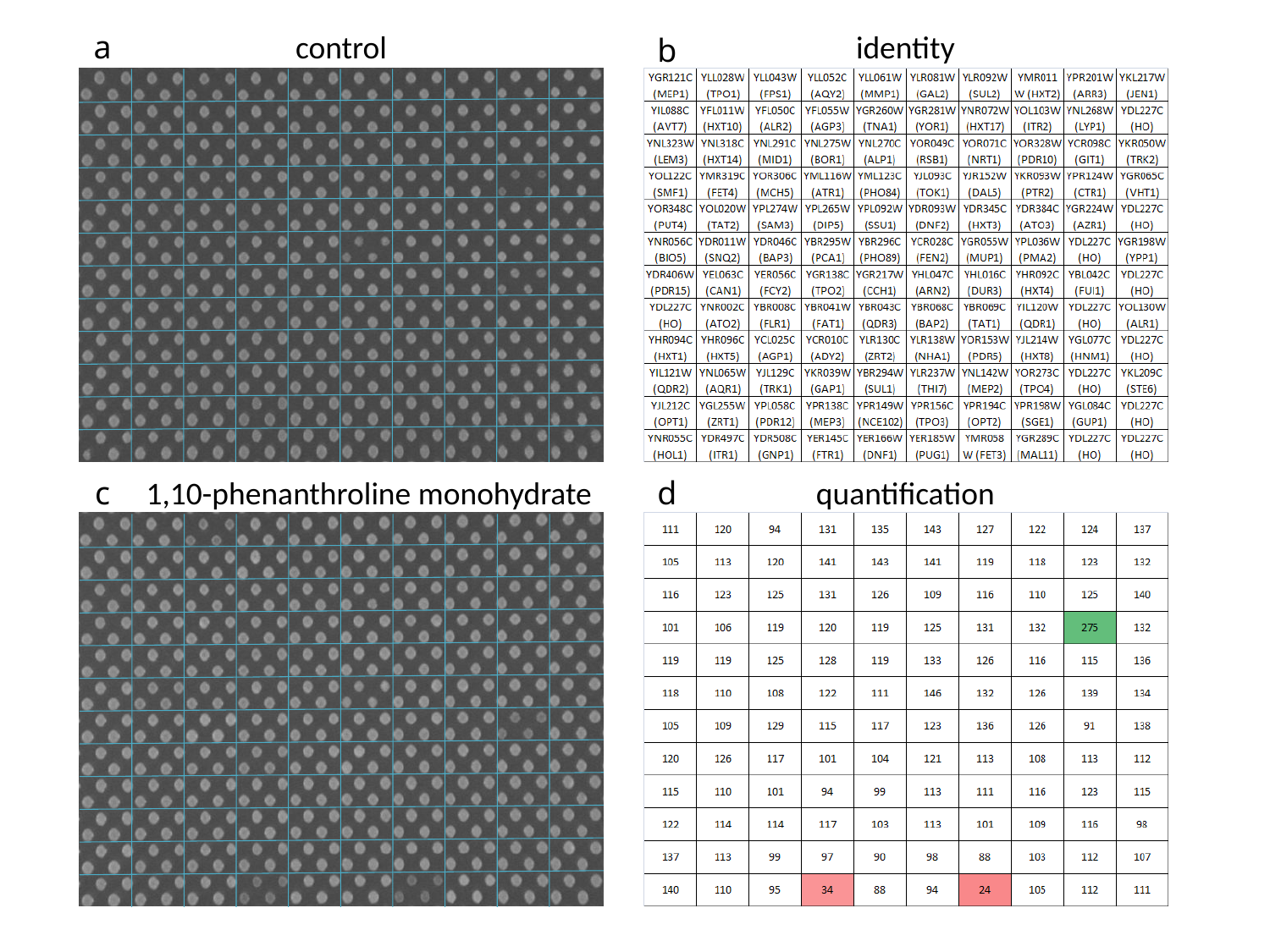

a
control
identity
b
c
1,10-phenanthroline monohydrate
d
quantification

Supplement: Additional file 22 — Identification of putative 1,10-phenanthroline transporters by the robot-assisted experiment. (a) Control plate with homozygous deletion mutant strains spotted in quadruplicates (by a Singer RoToR© HAD robot) onto F1 medium agar plate containing 1% DMSO. (b) Identity of deletion mutants spotted onto control and drug plates. (c) Homozygous deletion mutant strains spotted in quadruplicates (by a Singer RoToR© HAD robot) onto F1 medium agar plate containing 14 μM 1,10-phenanthroline. (d) Quantification of the relative growth between drug and control plates with 1,10-phenanthroline resistant strains highlighted in green. [file 1741-7007-9-70-S22.PPT]

## Slide 1
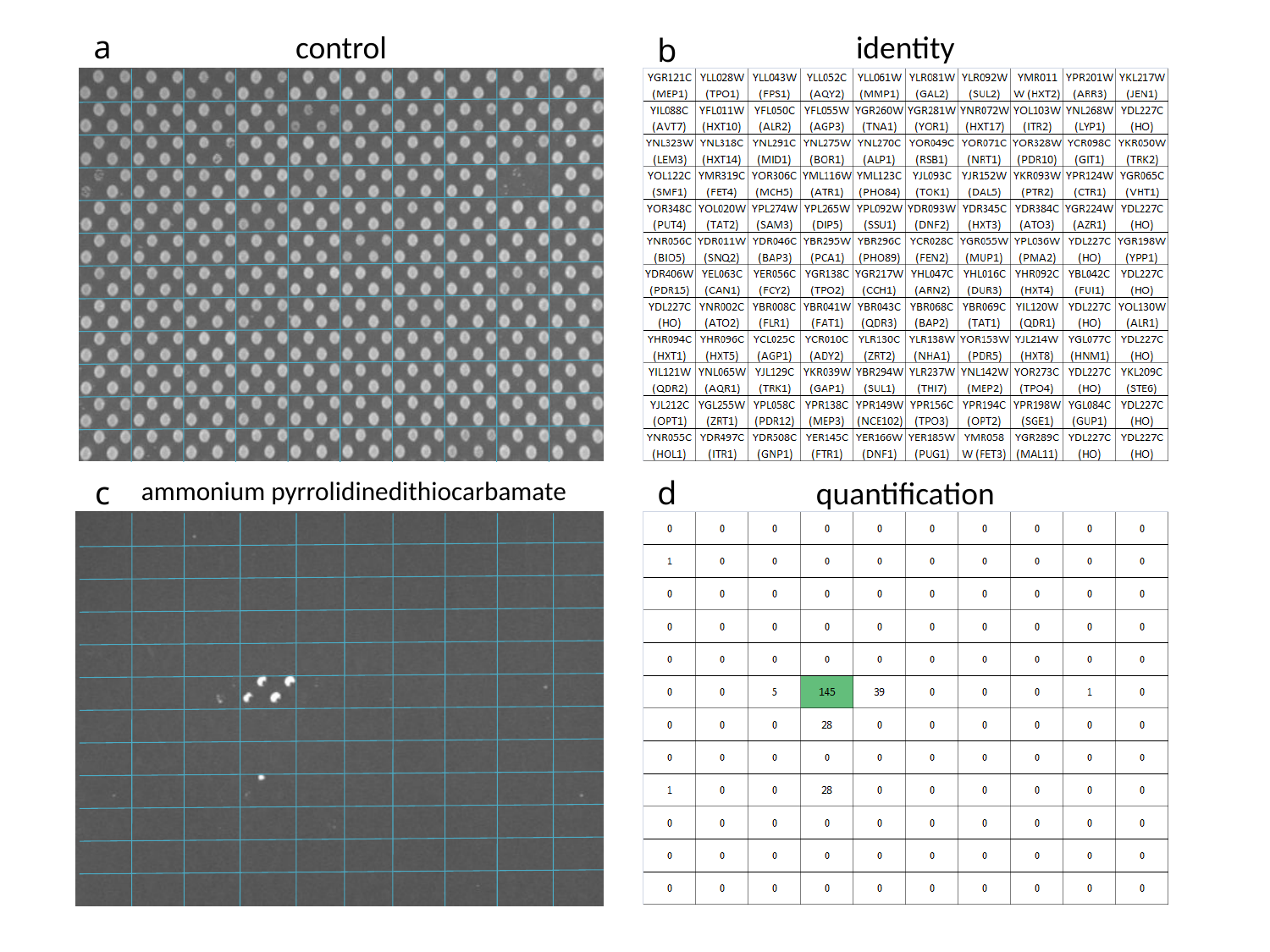

a
control
identity
b
c
d
quantification
ammonium pyrrolidinedithiocarbamate

Supplement: Additional file 23 — Identification of putative ammonium pyrrolidine dithiocarbamate transporters by the robot-assisted experiment. (a) Control plate with homozygous deletion mutant strains spotted in quadruplicates (by a Singer RoToR© HAD robot) onto F1 medium agar plate containing 1% DMSO. (b) Identity of deletion mutants spotted onto control and drug plates. (c) Homozygous deletion mutant strains spotted in quadruplicates (by a Singer RoToR© HAD robot) onto F1 medium agar plate containing 20 μM ammonium pyrrolidine dithiocarbamate. (d) Quantification of the relative growth between drug and control plates with ammonium pyrrolidine dithiocarbamate resistant strains highlighted in green. [file 1741-7007-9-70-S23.PPT]
