# Supplementary material for: Genome-wide assessment of the carriers involved in the cellular uptake of drugs: a model system in yeast
Source: BMC Biol. 2011 Oct 24;9:70. doi: 10.1186/1741-7007-9-70 (PMC3280192; doi:10.1186/1741-7007-9-70)
Supplement: Additional file 14 — Chemical structure of tunicamycin linked to the proposed transporters. [file 1741-7007-9-70-S14.PPT]

## Slide 1
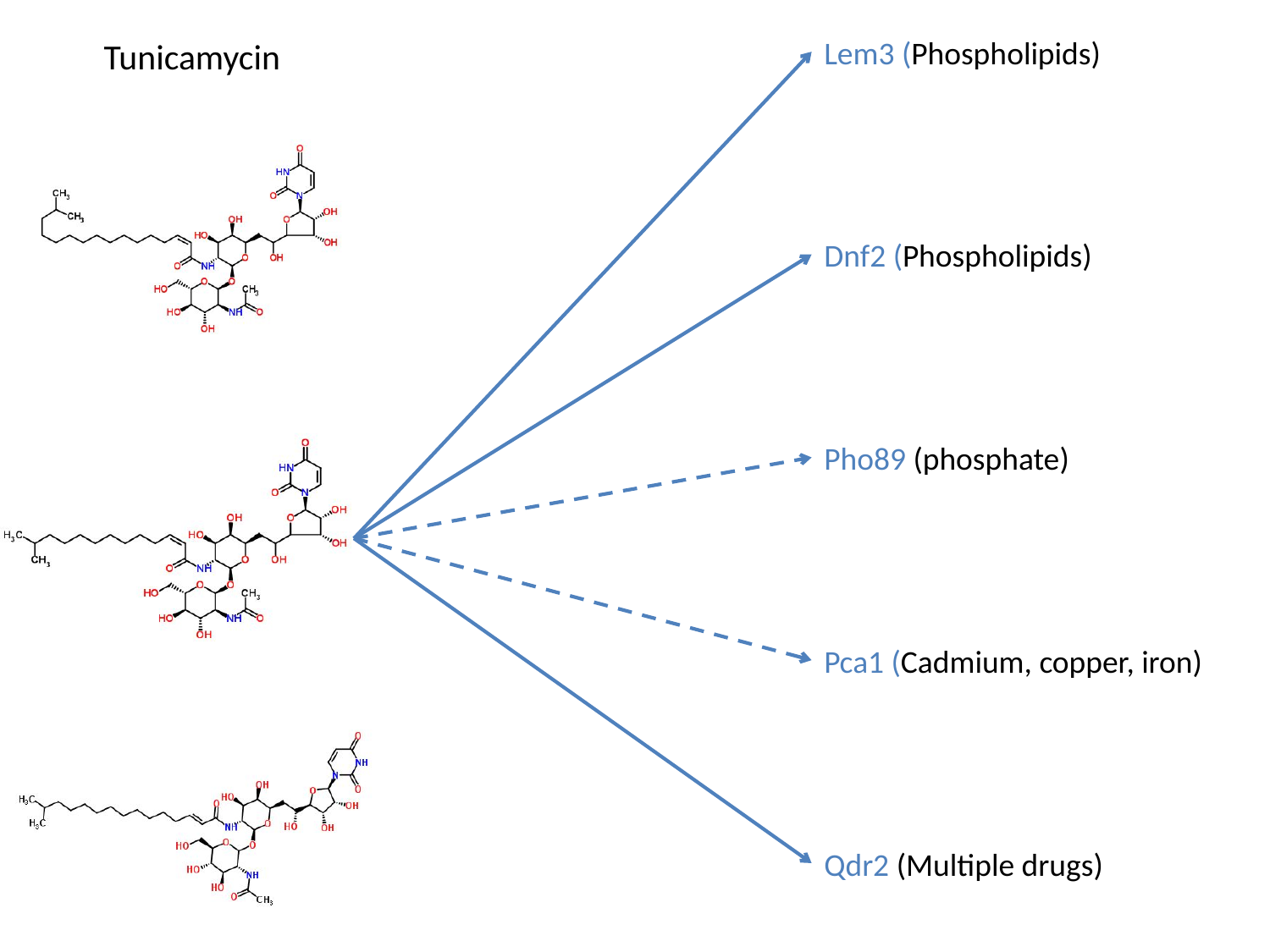

Lem3 (Phospholipids)
Tunicamycin
Dnf2 (Phospholipids)
Pho89 (phosphate)
Pca1 (Cadmium, copper, iron)
Qdr2 (Multiple drugs)
