# Supplementary material for: Genome-wide assessment of the carriers involved in the cellular uptake of drugs: a model system in yeast
Source: BMC Biol. 2011 Oct 24;9:70. doi: 10.1186/1741-7007-9-70 (PMC3280192; doi:10.1186/1741-7007-9-70)
Supplement: Additional file 24 — F1 minimal medium. Components I, II and III can be made up together at 5× final concentration and autoclaved. Component III can be made up at 5× final concentration and autoclaved. Component IV (vitamin solution) is filter-sterilized and kept at -20°C; aliquots are added to fresh 1× solution. Component V is made up as 40% w/v stock solution and autoclaved. [file 1741-7007-9-70-S24.PDF]

	Component
I	NH_4SO_4<br>KH_2PO_4<br>$MgSO_4.7H_2O$<br>NaCl<br>$CaCl_2.2H_2O$<br>--<br>Uracl<br>Histidine<br>Leucine
II	$ZnSO_4.7H_2O$<br>$CuSO_4.5H_2O$<br>H_3BO_3<br>KI
II	$FeCl_3.6H_2O$
IV	Inositol<br>Thiamine/HCL<br>Pyridoxine<br>Ca-pantothenate<br>Biotin
V	Glucose (from a 40% conc solution) (1)<br>glucose

*

**

***

****

*****
